# Using noninvasive metagenomics to characterize viral communities from wildlife

**DOI:** 10.1111/1755-0998.12946

**Published:** 2018-10-19

**Authors:** Laura M. Bergner, Richard J. Orton, Ana da Silva Filipe, Andrew E. Shaw, Daniel J. Becker, Carlos Tello, Roman Biek, Daniel G. Streicker

**Affiliations:** ^1^ Institute of Biodiversity, Animal Health and Comparative Medicine University of Glasgow Glasgow UK; ^2^ MRC–University of Glasgow Centre for Virus Research Glasgow UK; ^3^ Odum School of Ecology University of Georgia Athens Georgia; ^4^ Center for the Ecology of Infectious Diseases University of Georgia Athens Georgia; ^5^ Department of Microbiology and Immunology Montana State University Bozeman Montana; ^6^ Association for the Conservation Development of Natural Resources Lima Peru; ^7^ Yunkawasi Lima Peru

**Keywords:** *Desmodus rotundus*, microbial community, shotgun metagenomics, virome

## Abstract

Microbial communities play an important role in organismal and ecosystem health. While high‐throughput metabarcoding has revolutionized the study of bacterial communities, generating comparable viral communities has proven elusive, particularly in wildlife samples where the diversity of viruses and limited quantities of viral nucleic acid present distinctive challenges. Metagenomic sequencing is a promising solution for studying viral communities, but the lack of standardized methods currently precludes comparisons across host taxa or localities. Here, we developed an untargeted shotgun metagenomic sequencing protocol to generate comparable viral communities from noninvasively collected faecal and oropharyngeal swabs. Using samples from common vampire bats (*Desmodus rotundus*), a key species for virus transmission to humans and domestic animals, we tested how different storage media, nucleic acid extraction procedures and enrichment steps affect viral community detection. Based on finding viral contamination in foetal bovine serum, we recommend storing swabs in RNAlater or another nonbiological medium. We recommend extracting nucleic acid directly from swabs rather than from supernatant or pelleted material, which had undetectable levels of viral RNA. Results from a low‐input RNA library preparation protocol suggest that ribosomal RNA depletion and light DNase treatment reduce host and bacterial nucleic acid, and improve virus detection. Finally, applying our approach to twelve pooled samples from seven localities in Peru, we showed that detected viral communities saturated at the attained sequencing depth, allowing unbiased comparisons of viral community composition. Future studies using the methods outlined here will elucidate the determinants of viral communities across host species, environments and time.

## 1 INTRODUCTION

Microbial communities of bacteria and viruses play important roles in ecosystem function (Strickland, Lauber, Fierer, & Bradford, 2009; Strom, 2008; Suttle, 2007; van der Heijden, Bardgett, & Straalen, 2008) and in maintaining the health of organisms (Ley, Turnbaugh, Klein, & Gordon, 2006; Manrique et al., 2016; Muegge et al., 2011). Despite the importance of studying microbial communities in the environment and within hosts, classical methods of microbe discovery are not easily applied at the community level. For example, characterization by isolation and culturing is unsuitable for members of the microbial community that is difficult to grow in culture (Fancello, Raoult, & Desnues, 2012). Serological tests of antibody presence are targeted towards specific taxa and can be difficult to interpret due to antibody cross‐reactivity and inconsistent cut‐off thresholds for positivity (Gilbert et al., 2013). Molecular detection of nucleic acids by targeted PCR remains an important technique for sequencing specific genomic regions, but these approaches cannot identify all taxa present and are inappropriate for discovering new, highly divergent taxa as designing primers or probes requires prior knowledge of nucleotide sequences (Fancello et al., 2012; Temmam, Davoust, Berenger, Raoult, & Desnues, 2014). In contrast, unbiased deep sequencing has the potential to capture a snapshot of microbial communities in a large number of samples without prior expectations about what taxa will be detected.

Deep sequencing has illuminated the structure and function of microbial communities across time and space in ways that would not have been possible using traditional methods. In the field of ecology, theories developed at macro‐organismal level have been tested in microbial communities, such as the cycling of predator and prey populations (Rodriguez‐Brito et al., 2010) and the existence of elevational diversity gradients (Fierer et al., 2011). Deep sequencing has also demonstrated that both bacterial and viral communities differ across abiotic environments (Dinsdale et al., 2008) in such diverse systems as soil bacteria (Fierer et al., 2012) and marine viruses (Hurwitz, Westveld, Brum, & Sullivan, 2014). In the context of human and animal health, deep sequencing can identify candidate pathogens in unexplained disease (Briese et al., 2009; Cox‐Foster et al., 2007; Honkavuori et al., 2008; Palacios et al., 2008) and potential hosts and vectors of emerging pathogens (Masembe et al., 2012; Veikkolainen, Vesterinen, Lilley, & Pulliainen, 2014; Volokhov et al., 2017). Studies of host‐associated microbial communities have revealed that microbes vary across body habitats, space and time (Blekhman et al., 2015; Costello et al., 2009), and that a community‐level perspective of host‐associated microbes is critical for understanding health and disease (Lecuit & Eloit, 2013; Vayssier‐Taussat et al., 2014; Virgin, 2014). Sequencing host‐associated bacterial communities in wildlife has revealed that communities vary over time (Bobbie, Mykytczuk, & Schulte‐Hostedde, 2017), that social interactions are key determinants of community composition (Grieneisen, Livermore, Alberts, Tung, & Archie, 2017; Tung, Barreiro, Burns, & Grenier, 2015) and that dietary changes due to habitat degradation can alter bacterial communities (Amato et al., 2013). While host‐associated viral communities in wildlife remain relatively unexplored, the divergent responses of host‐associated bacteria and viruses to experimental diet modification (Howe et al., 2015) and the biological differences between the two types of microbes suggest that viral communities in wildlife might exhibit different patterns to those observed in bacteria.

Deep sequencing studies of microbial communities typically employ either metagenomics, which is the random sequencing of genomic fragments of an entire sample, or metabarcoding, which is a sequence‐specific PCR‐based approach (Creer et al., 2016). Studies of bacterial communities frequently use 16S ribosomal RNA (rRNA) metabarcoding to examine highly multiplexed samples. However, viral communities lack a similarly conserved marker across or even within viral families (Mokili, Rohwer, & Dutilh, 2012; Rohwer & Edwards, 2002) and are therefore more commonly characterized using metagenomics. Although this approach is currently less cost‐ and time‐efficient than metabarcoding for large numbers of samples, it can assign taxa at higher resolution (depending on factors such as read length, genomic region and reference database) and avoids PCR biases (Jovel et al., 2016). Shotgun metagenomics also allows the simultaneous characterization of different microbial communities (e.g., bacterial and viral) (Chandler, Liu, & Bennett, 2015; Schneeberger et al., 2016) as well as host population structure and diet (Srivathsan, Ang, Vogler, & Meier, 2016). Furthermore, metagenomics can detect viruses at or below the sensitivity of taxon‐specific PCR and qPCR (Greninger et al., 2010; Li et al., 2015; Yang et al., 2011), implying that broader taxonomic coverage does not necessarily trade off with sensitivity. Targeted approaches also likely underestimate or bias measures of viral diversity, potentially impacting downstream comparative analyses. The ability of metagenomics to sensitively detect taxa that are not specifically targeted and/or were previously undescribed has the potential to overturn prior understandings of viral community diversity and distribution based on serology and PCR.

Despite the great promise of metagenomics for studying viral communities, challenges inherent to sequencing viral genomes and technical uncertainties need to be addressed to maximize comparability. Viral communities include single‐ and double‐stranded viruses with both DNA and RNA genomes, ranging in size from 1,259,197 bp (*Megavirus chilensis;* Arslan, Legendre, Seltzer, Abergel, & Claverie, 2011) to 1,700 bp (*Hepatitis delta virus;* Taylor, 2006); larger viral genomes that have a higher probability of being sequenced may be over‐represented in the inferred community (Fancello et al., 2012). The RNA virus component of viral communities is highly sensitive to degradation due to temperature and storage conditions, raising questions about how samples should be preserved and transported. Indeed, different storage media alter viral detection in PCR‐based studies (Forster, Harkin, Graham, & McCullough, 2008; Osborne et al., 2011) and it is reasonable to assume the same in metagenomic studies.

Two popular methods for preserving viruses from field or clinical samples are viral transport media (VTM), an aqueous solution that typically contains protective proteins, antibiotics, and buffers to control the pH (Johnson, 1990) and RNAlater, a commercial reagent that penetrates tissues and stabilizes RNA (Ambion). VTM has historically been used to preserve samples when viruses are to be detected by PCR or cultured in vitro (e.g., Jensen & Johnson, 1994; Druce, Garcia, Tran, Papadakis, & Birch, 2012). Given the large number of historically collected samples in VTM, it would be ideal to include these in metagenomic studies. However, VTM may not be an appropriate medium because one commonly used component, foetal bovine serum (FBS), may be contaminated with bovine viruses. RNAlater is another popular medium for storing microbial samples collected in the field (Bányai et al., 2017; Drexler et al., 2011; Frick et al., 2017; Gomez et al., 2015), as it preserves RNA without requiring immediate freezing. However, its high salt content, while not problematic for solid tissue samples, creates challenges for nucleic acid extraction from the kinds of noninvasive swab samples that are typical of ecological field studies (e.g., blood, urine, faeces and saliva). While viruses are often extracted from an aliquot of supernatant (Baker et al., 2013; Tse et al., 2012; Wu et al., 2012), extraction from the swab itself may be desirable for samples stored in RNAlater (Vo & Jedlicka, 2014). These extraction procedures need to be tested and optimized for more widespread use in noninvasive viral metagenomics.

Another challenge for viral metagenomics is that since genomes are sequenced at random, larger host and bacterial genomes are preferentially detected relative to smaller viral genomes (Nakamura et al., 2009; Yang et al., 2011). For this reason, samples are often enriched for viruses using methods including nuclease treatment, filtration of host/bacterial particles, density gradient centrifugation and removal of rRNA (Hall et al., 2014; Kleiner, Hooper, & Duerkop, 2015; Kohl et al., 2015). DNase treatment is a well‐established and effective method of enrichment (Allander, Emerson, Engle, Purcell, & Bukh, 2001), while filtration and centrifugation are sometimes used but can bias the inferred viral community composition (Kleiner et al., 2015; Thurber, Haynes, Breitbart, Wegley, & Rohwer, 2009) and are impractical for ecological studies given the large numbers of samples typically processed and interest in generating community data rather than focusing on a particular pathogen. Depletion of host rRNA is unlikely to bias the viral community (He et al., 2010; Matranga et al., 2014), but may affect the distribution of coverage across the viral genome (Li et al., 2016). Identifying a combination of laboratory methods that maximize the proportion of viral reads while minimizing bias would allow greater multiplexing, enabling metagenomic studies of viral communities on an ecological or evolutionary scale.

Here, we describe a field–laboratory–bioinformatic pipeline to characterize viral communities in noninvasively collected faecal and oropharyngeal swabs from common vampire bats (*Desmodus rotundus*) in Peru. To optimize our protocol for comparative viral metagenomics, we first address the following questions: (a) Are samples stored in VTM containing FBS appropriate for viral metagenomics? (b) what is the most effective way to extract viral nucleic acid from swabs stored in RNAlater? and do the enrichment methods of (c) rRNA depletion and (d) DNase treatment increase the number of viral reads or viral taxa detected? Finally, we apply our optimized protocol to field‐collected samples to validate whether viral communities are reliably characterized at commonly attained depths of sequencing.

## 2 MATERIALS AND METHODS

### 2.1 Authorizations

Bat capture and sampling methods were approved by the Research Ethics Committee of the University of Glasgow School of Medical Veterinary and Life Sciences (Ref081/15) and the University of Georgia Animal Care and Use Committee (A2014 04–016‐Y3‐A5). Bat capture and sampling were approved by the Peruvian Government under permits RD‐009–2015‐SERFOR‐DGGSPFFS, RD‐264–2015‐SERFOR‐DGGSPFFS and RD‐142–2015‐SERFOR‐DGGSPFFS. Access to the genetic resources of Peru was granted under permit RD‐054–2016‐SERFOR‐DGGSPFFS.

### 2.2 Field sampling of common vampire bats

**Figure 1:**
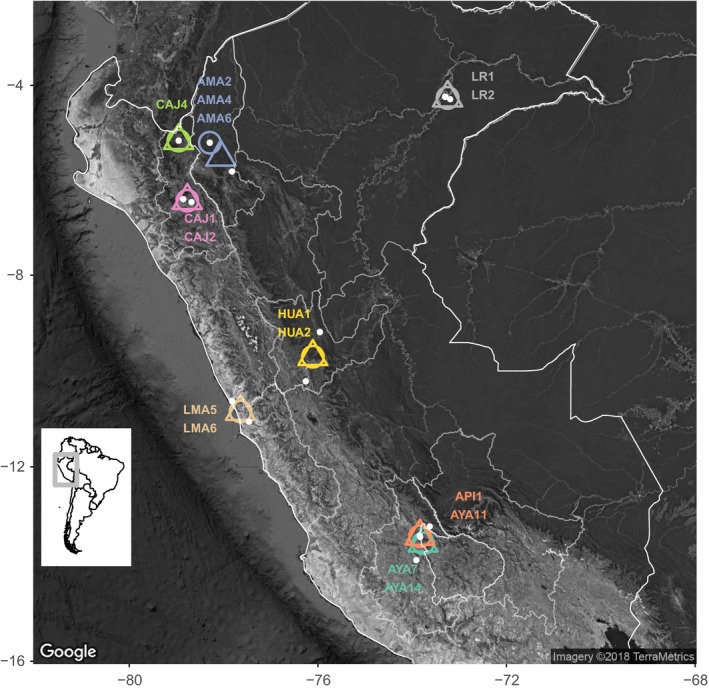
Sampling of vampire bat colonies used for enrichment and subsampling tests. Individual colonies are represented as white points and midpoints for each pool, in which one to two colonies were combined, are represented as circles (faeces) or triangles (saliva). Colony names are shown in the same colour as the pools in which they are included. Peru country borders and departments within Peru where samples were collected are outlined in white. The inset map shows South America, with Peru highlighted in the grey box [Colour figure can be viewed at wileyonlinelibrary.com]

Common vampire bats were captured at 16 sites in seven departments (administrative regions) across Peru (Figure 1) between 2015 and 2016. Roosts were either natural (caves, trees) or man‐made structures (abandoned houses, tunnels, mines) inhabited by bats. Bats were captured within roosts using hand nets or while they exited roosts using mist nets and harp traps. For nocturnal captures, nets were open from approximately 18:00–6:00 and checked every 30 min; a combination of one to three mist nets and one harp trap was used depending on the size and number of roost exits identified. When exact roost locations were unknown, bats were captured while foraging at livestock pens using mist nets. Upon capture, bats were placed into individual cloth holding bags before being processed and sampled. Bats were also given a uniquely numbered wing band (3.5 mm incoloy, Porzana Inc) for identification of recaptures in ongoing longitudinal studies.

Oropharyngeal (saliva) samples were collected by allowing bats to chew on cotton‐tipped wooden swabs (Fisherbrand) for 10 s. Faecal samples were collected by rectal swab, using a 3‐mm diameter rayon‐tipped aluminium swab (Technical Service Consultants Ltd) dipped in sterile Dulbecco's phosphate‐buffered saline DPBS (Gibco). Swabs were stored in uniquely numbered cryovials containing 1 ml RNAlater (Ambion) or VTM (10% foetal bovine serum, penicillin‐streptomycin, fungizone antimycotic). Following manufacturer's instructions, swabs in RNAlater were stored overnight at 4°C before being transferred to dry ice (ca. −80°C), while those in VTM were immediately placed on dry ice. Both were permanently stored in −70°C freezers.

### 2.3 RNA extraction

Unless otherwise noted, nucleic acid extractions were performed on a Kingfisher Flex 96 automated extraction machine (Thermo) with the BioSprint One‐For‐All Vet Kit (Qiagen) using a modified version of manufacturer's protocol for purifying viral nucleic acids from swabs (details in Supporting Information Appendix S1).

### 2.4 Bioinformatic analysis of viral communities

We created a bioinformatic pipeline for virus discovery and viral community analyses in shotgun metagenomic data from vampire bat samples (Supporting Information Appendix S2: Figure S1). Briefly, the pipeline filtered out low‐quality reads and duplicates, and then filtered out non‐viral reads including those matching the vampire bat genome (Zepeda Mendoza et al., 2018; NCBI BioProject Accession PRJNA414273), the PhiX Illumina sequencing control, ribosomal RNA and other reads with high matches to prokaryote/eukaryote sequences. Remaining reads were assembled into contigs, and then, both raw reads and assembled contigs were assigned to viral taxa by comparison with the ncbi viral refseq database.

Viral reads and contigs were converted into lists of viral taxa at different taxonomic levels using MEGAN Community Edition (Huson et al., 2016) with the default parameters of the lowest common ancestor (LCA) assignment algorithm, except that minimum score and minimum support per cent were set to zero to include all hits passing the filters of the bioinformatic pipeline (maximum *e*‐value of 0.001 for each Diamond blast step). For read‐level analysis, we did not consider species‐level assignments to be trustworthy as reads were only 150 bp long and could match equally well to numerous species within a genus. We included genera that are not yet assigned to families and species that are not yet assigned to genera. Taxa lists were filtered for vertebrate‐infecting viruses using a list of vertebrate‐infecting viral families and genera (Supporting Information Table S1) that was compiled from the 2017 ICTV Taxonomy (Adams et al., 2017). Viral family and genus richness were calculated using the r package vegan (Oksanen et al., 2017; R Core Team, 2017).

### 2.5 Pilot study 1: Are samples stored in viral transport media appropriate for viral metagenomic analysis?

Total nucleic acid from two aliquots of FBS was extracted, library prepared and sequenced using a shotgun metagenomic approach (Supporting Information Appendix S3) to evaluate the presence of bovine viruses and to determine whether another storage medium, such as RNAlater, would be more appropriate. The resulting reads were processed through the bioinformatic pipeline (Supporting Information Appendix S2).

### 2.6 Pilot study 2: What extraction method for swabs stored in RNAlater maximizes nucleic acid?

This experiment used swabs that were inoculated with known concentrations of viral particles to identify the extraction method that maximized viral nucleic acid from swabs stored in RNAlater and to assess efficiency and repeatability of the extraction protocol. Swabs were designed to mimic samples collected from the field, with the caveat that they did not include host material (e.g., faeces and saliva), bacteria, parasites or the community of viruses expected to be present in field‐collected samples. These other components of samples could impact extraction and PCR efficiency, for example, by acting as a carrier to enhance RNA extraction or through the presence of compounds that can act as extraction or PCR inhibitors. However, rather than inoculate field‐collected swabs, in which differences between sample types or between pathogen communities could introduce uncontrolled variation, we opted for “clean” mock swabs that would allow us to evaluate differences in viral detection between extraction methods.

Extraction tests used Schmallenberg virus (SBV), a single‐stranded RNA orthobunyavirus (Hoffmann et al., 2012). A 3.9 × 10^5^ plaque‐forming units (PFU)/ml stock of SBV was serially diluted in sterile Dulbecco's phosphate‐buffered saline DPBS (Gibco), and 10 μl of cell‐free virus at a range of dilutions from 10^6^ to 10^3^ copies/ml was inoculated into the same swabs used in field studies (Fisherbrand; Technical Service Consultants Ltd). Swabs were stored in 1 ml of RNAlater at −80°C overnight. We then extracted RNA from swabs using a manual approximation of the Kingfisher Flex 96 extraction method (Supporting Information Appendix S4), converted RNA to cDNA using random primers and quantified viral copy number using qPCR.

Our first test aimed to establish where in the sample the most extractable virus was located. RNA was extracted from three components of mock swabs (swab, supernatant and pellet). Three extraction replicates were performed for each component of swabs which had been inoculated at a concentration of 10^5^ copies/ml. All extraction replicates were quantified by qPCR in triplicate along with standards and no template controls (Supporting Information Appendix S4).

Our second test aimed to approximate the minimal detectable viral concentration by qPCR using this method, to assess repeatability and to estimate extraction efficiency using the cotton‐tipped wooden base swabs and rayon‐tipped aluminium base swabs used to collect samples in the field. RNA was extracted from swabs, converted into cDNA and quantified by qPCR as described above. Three extraction replicates were performed for each concentration from 10^6^ to 10^3^ copies/ml for cotton‐tipped wooden base swabs, and three extraction replicates were performed for aluminium base swabs at 10^5^ copies/ml.

### 2.7 Pilot study 3: Is rRNA depletion a useful enrichment method for characterizing viral communities?

We tested the effect of rRNA depletion on the number of viral reads and taxa detected. Swabs from 40 faecal and 10 saliva samples were extracted individually, quantified and pooled as described in Supporting Information Appendix S1. Five pools were created using nucleic acid extracts from the same sample type from 10 individuals across one to two sites in the same locality (between 0.14 and 74.1 km apart) within each department of Peru (Table 1; Figure 1).

**Table 1:** Multi‐colony pools sequenced for enrichment tests and subsampling. Pools were created by combining nucleic acid from 10 individual swabs of the same sample type from the same site or locality

Pool ID[Link]	Sample type	Raw reads	Viral reads	Colony 1[Link]	Colony 2[Link]	Test[Link]	Treatment	Subsampled
**AAC_H_F**	Faeces	12,166,001	10,870	AYA7	AYA14	–	–	Y
**AAC_H_SV**	Saliva	9,507,979	431	AYA7	AYA14	–	–	Y
**AAC_L_F**	Faeces	12,000,988	2,417	API1	AYA11	–	–	Y
**AAC_L_SV**	Saliva	15,121,355	609	API1	AYA11	–	–	Y
AMA_L_ F_NR	Faeces	17,827,799	2,062	AMA2	AMA6	rRNA	Non‐enriched	N
**AMA_L_F_R**	Faeces	17,760,709	28,344	AMA2	AMA6	rRNA	Enriched	N
**AMA_L_SV**	Saliva	9,363,273	305	AMA2	AMA4	–	–	Y
CAJ_L_F_NR	Faeces	15,940,753	1,179	CAJ4	–	rRNA	Non‐enriched	N
**CAJ_L_F_R**	Faeces	15,843,806	5,945	CAJ4	–	rRNA	Enriched	N
**CAJ_L_SV**	Saliva	8,685,456	600	CAJ4	–	–	–	Y
**CAJ_H_F_1**	Faeces	8,661,617	8,085	CAJ1	CAJ2	DNase	Light	Y
CAJ_H_F_2	Faeces	9,272,152	8,187	CAJ1	CAJ2	DNase	Harsh	Y
**CAJ_H_SV**	Saliva	11,830,542	534	CAJ1	CAJ2	–	–	Y
**HUA_H_F**	Faeces	10,814,816	11,285	HUA1	HUA2	–	–	Y
**HUA_H_SV**	Saliva	8,931,393	517	HUA1	HUA2	–	–	Y
LMA_L_F_NR	Faeces	19,605,605	1,425	LMA5	LMA6	rRNA	Non‐enriched	N
**LMA_L_F_R**	Faeces	17,365,381	8,206	LMA5	LMA6	rRNA	Enriched	N
LMA_L_SV_NR	Saliva	18,698,730	75	LMA5	LMA6	rRNA	Non‐enriched	N
**LMA_L_SV_R**	Saliva	15,953,442	483	LMA5	LMA6	rRNA	Enriched	N
LR_L_F_NR	Faeces	19,531,234	1,535	LR1	LR2	rRNA	Non‐enriched	N
**LR_L_F_R**	Faeces	13,843,629	4,544	LR1	LR2	rRNA	Enriched	N
**LR_L_SV**	Saliva	9,023,821	478	LR1	LR2	–	–	Y

All pool IDs reflect the locality (AAC, Ayacucho‐Apurímac‐Cusco; AMA, Amazonas; CAJ, Cajamarca; HUA, Huánuco; LMA, Lima; LR, Loreto) and sample type (F, faeces; SV, saliva). Some IDs also reflect elevation (H, high; L, low) to differentiate localities with multiple pools. NR and R correspond to ribosomal treatment, either non‐enriched or enriched, and one sample (CAJ_H_F) has associated numbers (1 and 2) referring to two batches that received different treatments during viral enrichment. Pools processed using the final protocol are shown in bold.

Colony codes correspond to department within Peru. Colony locations and pool midpoints are shown in Figure 1.

Enrichment tests are abbreviated as rRNA (ribosomal RNA depletion) and DNase (light or harsh DNase treatment).

Pools were treated with DNase I (Ambion); buffer and enzyme were scaled such that all reactions contained 1× DNase buffer and 2U DNase per 100 μl. Reactions were incubated at 37°C for 5 min, then cleaned up with 1.8× Agencourt RNAClean XP beads, eluted in RNase‐free water and split in half. Half of each DNase‐treated pool was enriched by rRNA depletion using the Ribo‐Zero rRNA Removal Kit (Human/Mouse/Rat) (Illumina) according to manufacturer's instructions, while the other half was library prepared directly, such that two libraries were prepared from each initial pool for a total of 10 libraries.

cDNA synthesis and library preparation were performed as described in Supporting Information Appendix S1 with a variable number of PCR cycles: Twelve cycles were used for non‐enriched samples, and 16 cycles were used for enriched samples. As rRNA depletion significantly decreased the quantity of nucleic acid, increased PCR cycles were necessary to generate sufficient material for sequencing for enriched samples; however, this difference is not expected to influence the proportion or composition of viral reads. Although PCR errors can be problematic in sequencing studies, we do not expect them to impact our results because viruses are assigned at the level of family or genus, rather than species or subspecies, where such errors could have a greater influence on taxonomic assignment. Final libraries were quantified, pooled, and sequenced (Supporting Information Appendix S1) and processed through the bioinformatic pipeline (Supporting Information Appendix S2).

### 2.8 Pilot study 4: Does intensive DNase treatment further enrich viral communities?

A harsher DNase treatment was also tested for its effect on the number of viral reads and viral taxa detected. Faecal swabs from 10 individuals across two sites in the Cajamarca Department (Table 1; Figure 1) were extracted and pooled (Supporting Information Appendix S1). The sample was split in half after pooling; one half was subjected to “light” treatment of 2U DNase and incubated at 37°C for 5 min (as above), and the other half was subjected to “harsh” treatment of 10U DNase and incubated at 37C for 15 min. Both halves were then cleaned up using a 1.8× ratio of Agencourt RNAClean XP beads. Following this step, pools were library prepared and sequenced according to the final protocol (Supporting Information Appendix S1) and processed through the bioinformatic pipeline (Supporting Information Appendix S2).

### 2.9 Subsampling analysis of viral community saturation using the optimized sequencing protocol

We conducted a subsampling analysis to test whether observed variation in the number of raw sequencing reads (Table 1) would affect the viral community detected (i.e., the number of viral reads, viral taxa and vertebrate‐infecting viral taxa). The data sets analysed included 12 multi‐colony pools (five faecal and seven saliva; Table 1) that had been sequenced according to our final protocol. Faecal and saliva pools contained swabs from individuals from the same colony or colonies, except in the Amazonas Department where saliva pools contained individuals from sites AMA2 and AMA4, but faecal pools contained individuals from sites AMA2 and AMA6. Subsampling comprised randomly selecting raw reads at every 10% between 10% and 100% of the total reads and was repeated five times per pool. Viruses from subsampled data sets were classified using the bioinformatic pipeline without the assembly step (Supporting Information Appendix S2).

A generalized linear mixed model (GLMM) with a Poisson distribution was used to assess the effect of the percentage of raw reads sampled on the number of viral taxa (families and genera) detected using the lme4 package of r (Bates, Mächler, Bolker, & Walker, 2015). Separate models were constructed for each combination of sample type (faecal and saliva), filtering condition (all viruses and vertebrate‐infecting) and taxonomic level (family and genus). The percentage of the total raw reads sampled was standardized by subtracting the mean and dividing by the standard deviation of percentages, and pool ID was included as a random effect in the model. For each data set, linear and second‐degree polynomial models were tested and compared using a likelihood ratio test and the change in Akaike information criterion (∆AIC), with a better fitting polynomial model indicating a plateau in the number of viral taxa detected at attained read depths.

## 3 RESULTS

### 3.1 Metagenomic sequencing reveals diverse viral nucleic acid in FBS (Pilot study 1)

A total of 21,501,182 raw reads were generated from the two batches of FBS. The bioinformatic pipeline detected 1,373 and 516 viral reads in each batch, respectively, which spanned 14 families of RNA and DNA viruses (Table 2). In both samples, the majority of viral reads were assigned to the family *Flaviviridae*, with 41% and 30% of viral reads for the two FBS batches, respectively, assigned to bovine viral diarrhoea virus 3 (BVDV‐3). Long contigs matching to BVDV (the longest were 1,396 and 775 bp, respectively, out of a full genome around 12,000 bp) had 96%–98% identity to strain SV757/15 of BVDV‐3 (Supporting Information Table S2 and S3).

**Table 2:** Viral families detected from shotgun metagenomic sequencing of FBS. For each viral family, the number of reads and contigs is reported for each of the two batches of FBS that were analysed

Family	FBS1[Link]		FBS2[Link]	
	Reads	Contigs	Reads	Contigs
*Adenoviridae*	27	0	40	2
*Asfarviridae*	2	0	0	0
*Myoviridae*	52	2	10	0
*Podoviridae*	29	0	47	5
*Siphoviridae*	73	4	32	2
*Herpesviridae*	2	2	6	1
*Iridoviridae*	1	0	0	0
*Polydnaviridae*	4	0	0	0
*Poxviridae*	9	0	0	0
*Retroviridae*	180	20	104	15
*Microviridae*	2	0	2	0
*Nyamiviridae*	0	0	1	0
*Flaviviridae*	950	15	267	11
*Alphaflexiviridae*	8	0	0	0
Total viral reads[Link]	1,373		516	
Raw reads	13,565,793		7,935,389	

FBS1 and FBS2 were two different batches of FBS that were sequenced.

Number of reads assigned to families do not add up to the total number of viral reads as some were classified as viral but not assigned to a family.

### 3.2 Viral sequences are maximized by extracting RNA from intact swabs (Pilot study 2)

For swabs stored in RNAlater, extracting directly from the swab itself yielded viral nucleic acid that was measurable by qPCR, while supernatant and pellet did not (data not shown). The limit of detection occurred with swabs that were initially inoculated with 220 viral copies; at this level, virus was inconsistently detectable by qPCR (Table 3). Virus became consistently detectable at 2,200 copies inoculated into the swab. Of the three aluminium‐base swabs that were inoculated with 2,200 copies, two of the extractions contained undetectable virus in all three qPCR replicate reactions, potentially because these swabs were smaller, and it was difficult to determine whether the virus had absorbed into the rayon. However, the one aluminium‐base swab with measurable virus was comparable in final copy number to the wooden‐base swabs (Table 3). The qPCR replicates were generally consistent aside from samples on the edge of detectability, but Ct and copy number varied between extraction replicates of swabs containing the same initial quantity of virus. For the swabs inoculated with 2,200 copies (aluminium and wooden‐base), there were on average 1,230 copies present following RNA extraction, yielding an extraction efficiency of about 56% (there were 1,578 copies and 72% efficiency when excluding an outlier wooden‐base swab replicate that had 0.94 qPCR copies).

**Table 3:** Summary of mock swabs tested for different extraction methods using qPCR. Swabs were inoculated with Schmallenberg virus and final virus concentration following extraction was measured using qPCR for different swab types and initial quantities of virus

Swab type	Virus concentration (copies/ml)[Link]	Initial swab quantity (copies)[Link]	Extraction Replicate	Average *C*_t_ (*SD*)	Average qPCR copies (*SD*)
Wooden‐base	10^4^	220	1[Link]	37.44 (0.45)	0.67 (0.20)
			2	No *C*_t_	No *C*_t_
			3[Link]	36.69 (0.72)	1.16 (0.55)
Wooden‐base	10^5^	2,200	1	33.86 (0.36)	7.84 (1.94)
			2	33.74 (0.6)	8.83 (3.79)
			3	36.98 (0.57)	0.94 (0.37)
Aluminium‐base	10^5^	2,200	1	34 (0.12)	7 (0.59)
Wooden‐base	10^6^	22,000	1	33.49 (0.35)	10.13 (2.4)
			2	31.72 (0.13)	33.70 (2.86)
			3	32.92 (0.32)	14.90 (3.06)

Notes

*SD:* standard deviation.

Indicates only two of the three qPCR replicates were measurable (one replicate was below the limit of detection). When all three qPCR replicates were below the limit of detection, this is indicated with no *C*_t_. All other average Ct and average qPCR quantities are calculated based on three qPCR replicates.

Virus concentration and initial swab quantities are calculated based on qPCR measurements of undiluted virus, which was then diluted to obtain the concentrations used in this experiment.

### 3.3 Viral enrichment is improved by rRNA depletion (Pilot study 3)

**Figure 2:**
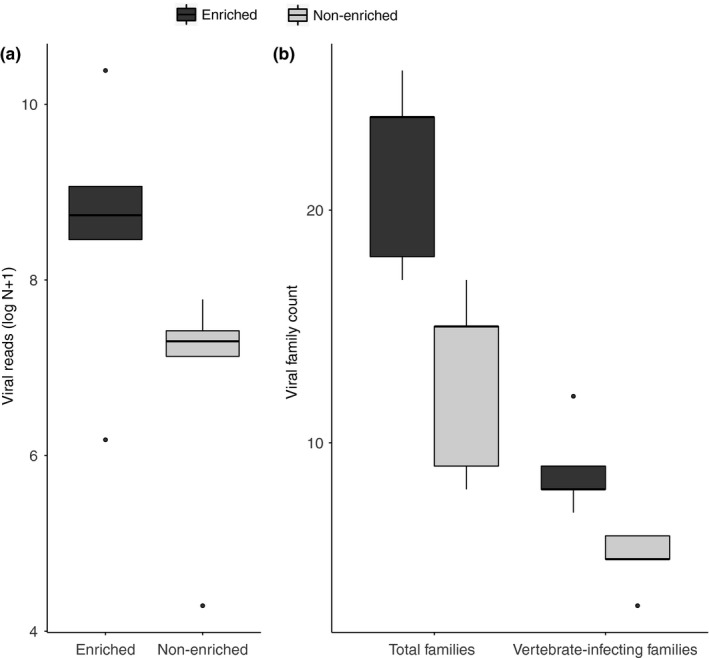
Comparison of viral reads and viral families in ribosomal depletion enrichment treatments. (a) Comparisons are shown for number of viral reads as log [*N* +1]) in enriched (*N* = 5) and non‐enriched (*N* = 5). (b) Total viral families and vertebrate‐infecting viral families detected in samples enriched by rRNA depletion (*N* = 5) compared to non‐enriched samples (*N* = 5)

**Figure 3:**
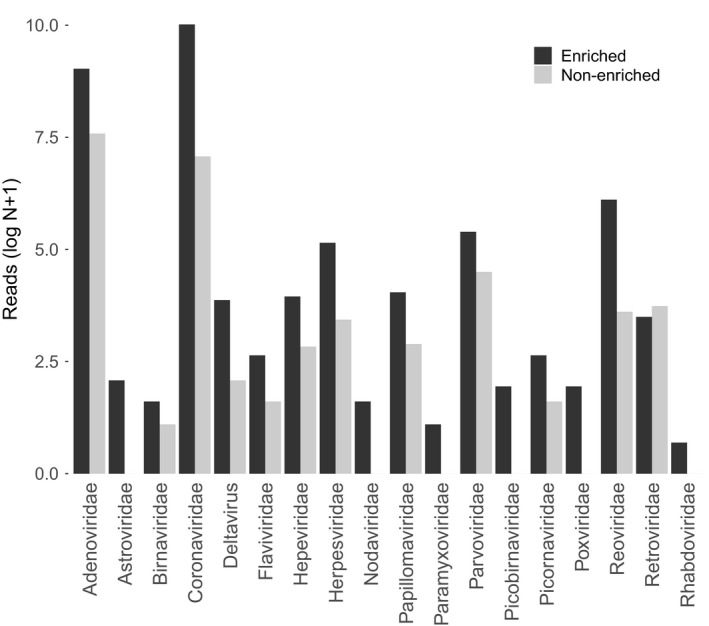
Comparison of reads per vertebrate‐infecting viral family across samples. Comparisons are shown for reads per vertebrate‐infecting viral family summed across samples enriched by ribosomal depletion (*N* = 5) and non‐enriched samples (*N* = 5). Read number comparison is shown for summed reads (as opposed to the mean) to enable visualization on a log scale

The sequenced faecal and saliva samples that were split and trialled for rRNA depletion yielded a total of 172,371,088 reads which were fairly evenly distributed across samples (Table 1). Samples that were enriched contained on average 8,213 more viral reads (Figure 2a), with this difference being close to statistically significant (paired Wilcoxon signed‐rank test, *p* = 0.06) despite the small sample size (*N* = 10). On average, there were nine more viral families (paired Wilcoxon signed‐rank test, *p* = 0.058) and 3.8 more vertebrate‐infecting viral families (paired Wilcoxon signed‐rank test, *p* = 0.06) per sample in enriched samples (Figure 2b). Within vertebrate‐infecting viral families, the number of reads per family was higher in enriched samples with the exception of the family *Retroviridae* (Figure 3).

Vertebrate‐infecting viral families that were detected only after enrichment exhibited diverse genome composition and structure including positive sense, single‐stranded RNA (*Astroviridae, Nodaviridae*), negative sense, single‐stranded RNA (*Rhabdoviridae, Paramyxoviridae*), double‐stranded RNA (*Picobirnaviridae*) and double‐stranded DNA (*Poxviridae*). Similar patterns were observed for all viruses, not just those infecting vertebrates, and results were consistent when analyses were repeated at the level of viral genera (data not shown). In summary, the rRNA depletion results suggest that removal of host rRNA allowed detection of more viral taxa (Figure 2b) and improved the sequencing depth for detected viruses (Figure 3).

### 3.4 Viral enrichment is improved by light DNase treatment (Pilot study 4)

**Figure 4:**
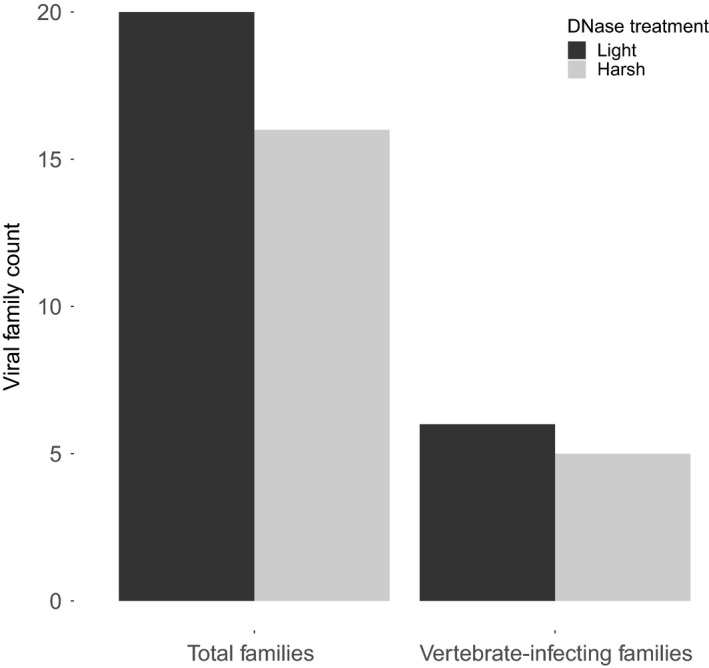
The number of total viral families and vertebrate‐infecting viral families detected after light and harsh DNase treatments. Comparisons show a single split sample, with half receiving light DNase treatment and half harsh DNase treatment

The faecal sample that was split and trialled for light/harsh DNase treatment yielded 17,933,769 reads that were evenly distributed across the two pools (Table 1). Although the number of viral reads was comparable between the two pools, light DNase treatment increased the taxonomic richness of viruses detected, both for all viruses and vertebrate‐infecting viruses (Figure 4).

The proportion of low complexity/PCR duplicate reads was also slightly higher in the harsh DNase treatment (1,974,128 reads) compared to the light treatment (1,620,909 reads) (Supporting Information Figure S2). suggesting that the harsh DNase treatment could have created a less diverse pool of nucleic acid prior to re‐amplification. Viral families that were absent in the harsh treatment included those with single‐stranded DNA genomes (*Circoviridae*), as well as single‐stranded RNA genomes (*Flaviviridae*), suggesting that DNase treatment may also degrade RNA viruses. However, RNA viruses were not always affected negatively by DNase treatment, as the single‐stranded RNA family *Paramyxoviridae* was present in the harsh treatment but not the light treatment. *Paramyxoviridae* was only represented by two reads in the harsh treatment so it could be a rare virus that was missing from the light treatment due to chance, but the effects of DNase on different viral genome types appear complex and may require more study to resolve. Although only two pools were compared, and they contained similar numbers of viral reads, a greater diversity of viral families was detected following the light DNase treatment.

### 3.5 Summary of samples sequenced using the optimized metagenomic protocol

Pooled samples processed according to the final protocol had similar numbers of raw reads, but the proportion of viral reads varied widely across samples (Table 1). Saliva samples consistently contained fewer viral reads than faecal samples. The proportion of reads filtered out during different stages of bioinformatic processing was fairly similar across samples (Supporting Information Figure S2), and we detected sequences matching to vertebrates, arthropods, bacteria and archaea in addition to the viral sequences that were the focus of our study (Supporting Information Figure S3).

### 3.6 Subsampling validates viral community saturation using the optimized protocol

**Figure 5:**
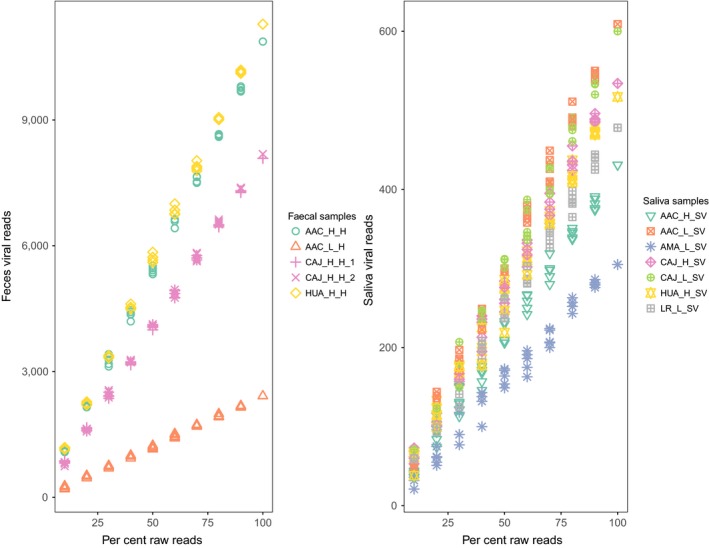
Viral reads increase proportionally to the percentage of raw reads analysed. The number of reads assigned as viral for faecal (*N* = 5) and saliva (*N* = 7) samples is shown at increasing percentages of total raw reads. Five replicates of each sample are depicted using the same symbol and colour; colours correspond to localities shown in Figure 1 [Colour figure can be viewed at wileyonlinelibrary.com]

**Figure 6:**
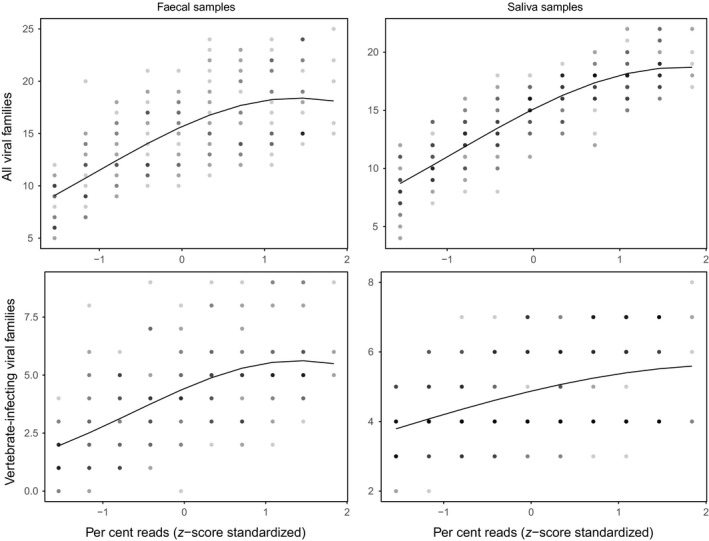
Viral communities saturate at high read depths. Panels show the number of viral families and vertebrate‐infecting viral families detected in faecal (*N* = 5) and saliva (*N* = 7) samples at increasing percentages of the total raw reads. Percentage reads are *z*‐score standardized by subtracting the mean and dividing by the standard deviation. Points, which are semi‐transparent to indicate density, show the rescaled original data and lines show the model prediction

The number of viral reads increased consistently with the number of raw reads, as would be expected with unbiased sequencing, though rate of increase differed among pools (Figure 5). In contrast, the number of viral families and vertebrate‐infecting viral families plateaued at higher percentages of the total number of raw reads sampled (Figure 6), and models explaining the number of viral families with a second‐degree polynomial effect of percentage of raw reads generally fit the data better than linear models (Table 4). The exception was vertebrate‐infecting viral families detected in saliva; however, detections did plateau at the viral genus level (Supporting Information Table S4; Figure S4). Aside from vertebrate‐infecting viral families in saliva, viral richness plateaued at around 80% of the total reads (Figure 6; Supporting Information Figure S4). Converting this to number of raw reads indicated that, on average, new detections began to level off at 8,358,626 reads (range: 6,929,294–12,097,084).

**Table 4:** Model comparison for viral family detection in subsampling analyses. Linear and polynomial models were compared for each sample type (faeces and saliva) and filtering (all viral families and vertebrate‐infecting only) combination at the family level. For each combination, two models were run and compared through both likelihood ratio test (*L*, χ^2^, *df* and *p*‐value) and AIC (AIC and ΔAIC)

	Model	*L*	χ^2^	*df*	*p‐*Value	AIC	ΔAIC
Faecal viral families	Linear	−556.1	17.271	1	3.24E−05	1,118.2	15.271
	Polynomial	−547.47				1,102.9	
Saliva viral families	Linear	−772.02	18.304	1	1.88E−05	1550	16.304
	Polynomial	−762.87				1533.7	
Vertebrate‐infecting faecal viral families	Linear	−407.39	10.356	1	0.00129	820.79	8.3564
	Polynomial	−402.22				812.43	
Vertebrate‐infecting saliva viral families	Linear	−573.15	0.8262	1	0.3634	1,152.3	1.174
	Polynomial	−572.73				1,153.5	

## 4 DISCUSSION

We developed a field–laboratory–bioinformatic protocol for characterizing viral communities, incorporating the following findings from pilot studies to maximize viral detections:

1. Swab samples should be stored in RNAlater rather than VTM containing FBS.
2. Nucleic acids should be extracted directly from swabs, rather than from supernatant or pellet.
3. Enrichment should use rRNA depletion and light DNase treatment.

The metagenomic pipeline yielded viral community data from swab samples taken from vampire bats across Peru, and detections in most cases plateaued within commonly attained levels of sequencing depth (Figure 6), suggesting that we developed an effective noninvasive method for sampling viral communities from field samples collected from wildlife. The field protocol standardizes sample collection, storage and transportation among geographically widespread and remote study sites. The laboratory and bioinformatic protocols aim to capture and identify as many different types of viruses as possible, while processing large batches of samples and avoiding well‐known sources of bias.

Metagenomic sequencing revealed diverse bovine viral nucleic acid in FBS. Importantly, our results are unlikely to indicate the presence of live viruses in FBS since commercial FBS is often heat‐inactivated and screened for live viruses. Instead, our detections probably represent viral nucleic acids which persist after heat inactivation, but which could nevertheless impact metagenomic studies. Detecting BVDV is unsurprising, as it is a common cell culture contaminant that has previously been found in high quantities in FBS (Allander et al., 2001; Gagnieur et al., 2014). Consistent with the South American origin of the FBS used in our analyses, BVDV‐3, or HoBi‐like viruses, was initially reported in FBS from South America and is likely endemic to livestock in Brazil (Bauermann & Ridpath, 2015). The consistent presence and proportion of BVDV as well as *Retroviridae* and several bacteriophage families (Table 2) across FBS batches suggest that this source of contamination could perhaps be accounted for in order to include VTM samples containing FBS in metagenomics studies. However, reads in FBS also matched the family *Adenoviridae* (genus *Mastadenovirus*), which are also common in bats (Drexler et al., 2011; Li et al., 2010), including neotropical species (Wray et al., 2016). If bat samples stored in VTM were sequenced and filtered for viral genera detected in FBS, this would potentially exclude true bat viruses. Our results therefore suggest that metagenomic results from historical samples stored in media containing FBS should be interpreted with caution and avoided where possible.

Our comparison of RNA extractions from different components of samples (swab, supernatant, pellet) showed that swab extraction, but not extraction from supernatant or pellet, typically yielded measurable nucleic acid. This could be due to the high salt concentrations in RNAlater that are designed to inhibit RNase activity, but which could also interfere with extraction from the supernatant/pellet. Typically, tissues stored in RNAlater are blotted to remove excess solution, and other samples such as blood are centrifuged, and the supernatant is removed prior to extraction. Unfortunately, it is not possible to completely remove the RNAlater from swabs but extracting from the swab itself might minimize salts relative to the other components of the sample. It is also possible that virus particles mostly remain within the swab itself when stored in RNAlater.

Direct extraction from swabs was previously used to characterize bacterial communities from swabs stored in RNAlater (Vo & Jedlicka, 2014), and other studies have released particles bound to swabs through incubation in lysis buffer (Schweighardt, Tate, Scott, Harper, & Robertson, 2014) or lysis buffer and proteinase K (Corthals et al., 2015; Ghatak, Muthukumaran, & Nachimuthu, 2013). We tested only one virus in this experiment, which may limit our ability to extrapolate the estimated limit of detection or extraction efficiency to other viruses with different characteristics or to field‐collected samples that include host cells and other material. In addition, the quantity of viral RNA extracted from swabs did not appear highly repeatable between extraction replicates. However, our results indicated that extracting directly from the swab improved viral detection relative to other components of the sample.

Our study tested a variety of laboratory methods for enhancing unbiased detection of viruses. The rRNA depletion results suggested that removing host rRNA increased both the number of viral reads and number of viral taxa detected without biasing the viral community, as has been observed in previous studies (He et al., 2010; Matranga et al., 2014). The only case in which there were more reads in the non‐enriched samples was the family *Retroviridae;* however, retroviruses integrate into the host genome and are likely to behave differently than other viral taxa with respect to enrichment. Although the Ribo‐Zero kit is described as being for human/mouse/rat and should be tested before use on other sample types, it has been used effectively on samples from taxa as distantly related as mosquitos (Weedall, Irving, Hughes, & Wondji, 2015), and we also found it to be effective for enriching samples taken from bats.

Although we were only able to analyse one split sample, the light DNase treatment results suggested an increase in the number of viral taxa detected compared to the harsher treatment. DNase is a well‐established method to reduce the number of host and bacterial reads relative to virus (Allander et al., 2001). Our light treatment was intended to knock down rather than remove all DNA, also potentially allowing for better detection of bacteria and parasites compared with an intensive enrichment, although we did not test this explicitly. Although this step could have caused bias towards RNA viruses, DNA virus reads occurred in all samples, as has been found in other viral metagenomic studies using an RNA‐based approach (Hall et al., 2014; Kohl et al., 2015; Wu et al., 2016), including those with a DNase treatment step (Baker et al., 2013; Hall et al., 2014). This could be explained by the presence of viral RNA transcripts, DNA viruses that replicate through an RNA intermediate (e.g., *Hepadnaviridae*), the ability of some DNA virus families to integrate into the genome of their host (e.g., *Herpesviridae*) or DNA being carried through the DNase treatment into library prep due to the light treatment or less than perfect efficiency of the reaction.

Although more intensive enrichment such as filtration or centrifugation could potentially have increased the number of viral reads, such methods are known to be biased against certain taxa (Conceição‐Neto et al., 2015; Kleiner et al., 2015; Wood‐Charlson, Weynberg, Suttle, Roux, & Oppen, 2015). In addition, it would be impossible to include a filtration step since swabs were immediately treated with lysis buffer in the extraction, leading to lysis of the viral particles which would normally be selected for using filtration. In the light of the above results, and despite the relatively small number of samples, we recommend rRNA depletion and light DNase treatment as an effective combination for viral enrichment.

It is worth noting the caveats of analysing noninvasively collected samples. First, although contamination has not been well characterized in viral metagenomic studies, it is a known problem in bacterial community studies. Samples with low microbial biomass are particularly sensitive to contamination with other microbes, for example from DNA extraction kits (Salter et al., 2014) or ultrapure water (Laurence, Hatzis, & Brash, 2014). Our protocol minimized this risk by pooling samples following extraction to increase the amount of target nucleic acid relative to potential reagent‐derived contaminants in downstream steps. Second, noninvasive samples will only detect viruses that are actively shed in urine and faeces, thus may miss latent viruses that are sporadically shed, but might be detectable by sequencing organs from sacrificed animals (Amman et al., 2012). Third, our protocol is not able to discriminate between viruses actively infecting hosts and transient viruses acquired from diet or the environment. Although some sources of bias are unavoidable, and it is likely that not all viral taxa in a given sample will be identified, the same is true of all studies in community ecology where exhaustive sampling is not possible (Gotelli & Colwell, 2001; Hughes, Hellmann, Ricketts, & Bohannan, 2001), and we showed statistically that viral communities in our samples were adequately sampled (Figure 6). Our approach yielded sufficient depth to confidently characterize viral communities at the viral family or genus level, while identification of species or strains might be achieved by further increasing read depths to generate longer contigs that could be more precisely assigned (Figure 5).

In summary, our pipeline simultaneously generated comparable viral communities from large numbers of noninvasively collected wildlife samples. A standardized approach to viral metagenomics opens many potential avenues of research in disease and community ecology. For example, viral community data collected across multiple individuals, populations and species allow the investigation of ecological processes shaping host‐associated viral community structure (Anthony et al., 2015; Olival et al., 2017). Taxonomic and functional patterns of bacterial diversity across host species are influenced by diet and phylogeny (Ley et al., 2008; Muegge et al., 2011; Zepeda Mendoza et al., 2018), but drivers of host‐associated viral communities may be different. In humans, host‐associated viral communities are stable over time within individuals, but highly variable between individuals (Minot et al., 2011; Reyes et al., 2010). These observations suggest the potential to use viral communities as a host or environmental “fingerprint” to evaluate interactions between multiple hosts, or between hosts and environments, as has been proposed in humans and primates (Fierer et al., 2010; Franzosa et al., 2015; Stumpf et al., 2016). Finally, although it was not the focus of our study, we also detected reads from vertebrates, protozoa and bacteria (Supporting Information Figure S3), suggesting that with appropriate bioinformatic modifications, shotgun metagenomic data generated using our protocol could simultaneously shed light on host genetics, diet, other non‐viral pathogens and commensal microbes. As metagenomics becomes an ever more popular and powerful tool for viral ecology, use of standardized methods such as those developed here will be crucial for comparative insights from diverse host species and environments.

## AUTHOR CONTRIBUTIONS

L.M.B., R.J.O., R.B. and D.G.S. conceived and designed the study. D.G.S., D.J.B. and C.T. coordinated field sampling and collected the samples. L.M.B., A.S.F. and A.E.S. designed and performed laboratory experiments. L.M.B., R.J.O. and D.G.S. analysed the data.

## Data Availability

Raw sequencing reads have been uploaded to the European Nucleotide Archive (ENA) under accession number PRJEB28138. Scripts described in Supporting Information Appendix S2 are available on GitHub (https://github.com/rjorton/Allmond).
