# Supplementary material for: Using noninvasive metagenomics to characterize viral communities from wildlife
Source: Mol Ecol Resour. 2018 Oct 19;19(1):128–43. doi: 10.1111/1755-0998.12946 (PMC6378809; doi:10.1111/1755-0998.12946)
Supplement: Supplementary file 1 [file MEN-19-128-s001.pdf]

Supporting Information for:

Using non-invasive metagenomics to characterize viral communities from wildlife

Laura M. Bergner, Richard J. Orton, Ana da Silva Filipe, Andrew E. Shaw, Daniel J. Becker, Carlos Tello, Roman Biek, Daniel G. Streicker

Table of Contents:

**NGS final protocol**	Page 1
**Bioinformatic pipeline**	Page 4
**FBS sequencing**	Page 5
**Mock viral swab extraction**	Page 6
**Tables**	Page 8
**Figures**	Page 18
**References**	Page 22

Appendix S1: NGS final protocol

Extraction

Total nucleic acid was extracted from samples using a Biosprint One-for-all Vet Kit (Qiagen) using a modified version of the manufacturer's protocol for purifying viral nucleic acids from swabs. Prior to inactivation, samples were processed in a MSC flow cabinet in a CL2+ laboratory. Extractions were performed by thawing samples and removing swabs using sterile forceps.

Each swab was first incubated in 144 µL Buffer RLT and 20 µL Proteinase K (both Qiagen) at 56°C for 15 minutes, vortexed for 15 seconds, then transferred to a second tube with 144 µL Buffer RLT and Proteinase K and incubated again for 15 minutes and vortexed again for 15 seconds. Samples were considered inactivated at this stage due to Buffer RLT, a lysis buffer containing guanidine isothiocyanate. The swab was then discarded and the two lysis buffer/Proteinase K solutions were briefly centrifuged, then combined and placed into a deep-well 96 sample extraction block with 25 µL MagAttract Suspension G (Qiagen) and 300 µL isopropyl alcohol. All of these steps were performed according to CL2+ guidelines.

Plates for wash steps were prepared; these included one plate containing 700 µL wash buffer AW1 and two plates containing 500 µL wash buffer RPE (both Qiagen). All plates were loaded onto a Kingfisher Flex 96 automated extraction machine (Thermo). The instrument settings, provided by Qiagen ('Protocol for purification of viral nucleic acid and bacterial DNA with Thermo Scientific KingFisher Flex'), consist of a lysis and binding step, followed by three wash

steps, and a final elution in 80 µL Buffer AVE (RNase-free water with 0.04% NaN_3). Extracted nucleic acid was stored at -80°C.

Pooling and viral enrichment
All samples were quantified using a Qubit 3.0 fluorometer and a Qubit RNA HS Assay (Life Technologies) to determine RNA concentration for pooling. Samples with measurable RNA were pooled at approximately 120 ng RNA and unmeasurable samples were pooled up to a maximum volume of 30 µL as possible. Pools were then treated with DNAse I (Ambion) to digest high molecular weight genomic DNA. Pool volume varied but buffer and enzyme were scaled such that all reactions contained 1X DNAse buffer and 2 Units (U) DNAse per 100 µL. Reactions were incubated at 37°C for 5 minutes, then immediately cleaned up with 1.8X Agencourt RNAClean XP beads (Beckman Coulter), washing the beads three times with 80% ethanol. Samples were then eluted in 20 µL nuclease-free water; 10 µL of eluate was used as input into the rRNA depletion step.

Pools were enriched by rRNA depletion using the Ribo-Zero rRNA Removal Kit (Human/Mouse/Rat) (Illumina) according to the manufacturer's instructions. Briefly, room temperature magnetic beads were prepared by washing twice in RNAse-free water, and then beads were resuspended in 65 µL Magnetic Bead Resuspension Solution. To each 10 µL RNA sample was added 18 µL RNAse-free water, 4 µL Ribo-Zero rRNA Reaction Buffer and 8 µL Ribo-Zero Removal Solution. Reactions were incubated at 68°C for 10 minutes, then at room temperature for 5 minutes to hybridize rRNA to probes. Pre-hybridized samples were added to the magnetic bead solution, then incubated at room temperature for 5 minutes followed by 50°C for 5 minutes. Samples were placed on a magnetic stand and 90 µL supernatant was removed, while beads containing hybridized rRNA were discarded. The enriched sample was cleaned up using 1.8X RNAClean XP beads and eluted in 10 µL RNAse-free water.

Library preparation
First strand cDNA synthesis was performed using the Maxima H Minus First Strand cDNA Synthesis Kit (Thermo) by incubating 10 µL nucleic acid with 1 µL dNTPs and 1 µL random hexamers at 65°C for 5 minutes. Reactions were chilled on ice and then 4 µL 5X reverse transcriptase buffer, 1 µL reverse transcriptase, and 3 µL PCR-grade water were added for a total reaction volume of 20 µL. Reactions were incubated on a thermocycler at 25°C for 10 minutes, 60°C for 45 minutes, and 85°C for 5 minutes. Single strand cDNA was then immediately converted to double strand cDNA using the NEBNext mRNA Second Strand Synthesis Module (New England Biolabs) by adding 8 µL 10X second strand synthesis buffer, 4 µL second strand synthesis enzyme, and 48 µL PCR-grade water for a total reaction volume of 80 µL. Reactions were incubated on a thermocycler at 16°C for 2.5 hours, and dsDNA was stored at -20°C until library preparation.

Samples were library prepared using the KAPA DNA Library Preparation Kit for Illumina (KAPA Biosystems) modified for low input RNA samples. DNA was first cleaned up with 80 µL (1:1 bead:sample ratio) of Ampure XP beads (Beckman Coulter). Samples were eluted in 52 µL 10 mM Tris (pH 8.5) and beads left in solution. End repair was performed by adding 6 µL 10X end

repair buffer and 2 µL end repair enzyme, and the 60 µL reaction was incubated at 20°C for 30 minutes. Samples were then cleaned up by adding 60 µL (1:1 ratio) of KAPA PEG/NaCl SPRI solution (KAPA Biosystems) to re-bind the DNA to the beads. Samples were eluted in 25 µL of Tris following clean-up, leaving the beads in solution.

A-tailing reactions were performed by adding 3 µL 10X A-tail buffer and 2 µL A-tail enzyme, then the 30 µL reaction volume was incubated at 30°C. 70 µL Tris was added to the reaction for a total volume of 100 µL, to which 100 µL SPRI solution (1:1 ratio) was added for cleanup. Samples were eluted in 15 µL Tris, leaving the beads in solution.

At this point, sample concentration was measured using a Qubit dsDNA HS Assay. Qubit readings were converted into picomoles (pmol) according to the formula (ng DNA * volume)/(average size (kbp) * 660). The amount of adapter added (pmol) was calculated as pmol DNA * 20, and for libraries with unmeasurable DNA, 0.5 µL of a 1:100 dilution of 15 µM adapter was added. Adapters used were NEBNext Adaptor for Illumina (New England Biolabs). Tris was added to calculated volume of adapters up to a total volume of 5 µL and this was combined with 14 µL A-tailed DNA, 5 µL 5X buffer and 1 µL T4 DNA ligase. The reaction was incubated at 20°C, then 1 µL USER enzyme was added to the reaction and incubated at 37°C to cleave a hairpin in the adapter. 74 µL Tris was added for a total volume of 100 µL and 100 µL SPRI solution was added for cleanup. Samples were eluted in 11 µL Tris, this time removing DNA from beads.

PCR re-amplification was performed by first combining 12.5 µL PCR mastermix with 10 µL DNA. Each sample was barcoded using 1.25 µL universal primer and 1.25 µL individually barcoded primer (NEBNext Multiplex Oligos for Illumina Index Primers Set 1, New England Biolabs) or 1.25 µL of two different individually barcoded primers (Dual Index Primers Set 1, New England Biolabs). Number of PCR cycles varied depending on DNA concentration, from 12 cycles for higher concentrations up to a maximum of 16 cycles for undetectable DNA. Thermocycling parameters were: 3 minutes at 95°C, 12-16 cycles of 20s at 98°C, 15s at 65°C, and 30s at 72°C, followed by 2 minutes at 72°C.

Following PCR, 75 µL Tris was added for a total volume of 100 µL. Then 90 µL Ampure beads (0.9:1 bead:sample ratio) was added; the smaller ratio is intended to eliminate small fragments in the library such as primer dimer and adapter dimer. After bead cleanup, samples were eluted in 15 µL Tris. Libraries were validated using a Qubit dsDNA HS Assay and TapeStation 4200 D5000 ScreenTape (Agilent). Post-PCR libraries were often found to have high molecular weight peaks which can affect calculations for pooling and loading the sequencing instrument; in this case, Ampure beads were used in a size selection step. A 0.6X ratio of Ampure beads was added to the samples and the supernatant was removed, with larger fragments being retained by the beads. Ampure beads were then added to the supernatant for a final ratio of 1.4X beads/PEG-NaCl to sample, and samples were eluted in 15 µL Tris. Final libraries were pooled in equimolar ratios, and validation of the final pool was performed using a Qubit dsDNA HS Assay and a TapeStation D1000 ScreenTape. Sequencing was performed on an Illumina NextSeq500 at the MRC-University of Glasgow Centre for Virus Research.

Appendix S2: Bioinformatic pipeline

Sequences were first demultiplexed according to barcode by the sequencing facility, then quality filtered using Trim Galore (Martin 2011; Andrews 2010). Low complexity reads and PCR duplicates were filtered out using prinseq-lite (Schmieder & Edwards 2011). Reads were mapped against the vampire bat genome (Mendoza et al. 2018) as well as the genome of the PhiX virus that is used as a positive control in Illumina sequencing, and is a widespread contaminant of previously published microbial genomes (Mukherjee *et al.* 2015). Mapping was performed using bowtie2 (Langmead *et al.* 2009), and only unmapped reads were retained for further analysis. The program RiboPicker (Schmieder *et al.* 2012) was then used to remove reads associated with rRNA, as some will be sequenced despite the rRNA depletion treatment.

We further used Diamond (Buchfink *et al.* 2014) to remove reads mapping to other eukaryotes and prokaryotes by comparing reads with the non-redundant NCBI database. Reads mapping to viruses by Diamond were retained for the next step of the analysis using a custom script Allmond (https://github.com/rjorton/Allmond) written for this purpose. Reads with no hits were also retained because they could be of viral origin, but not yet characterized in databases, or subsequently form larger contigs that can be classified as viral. The remaining reads (viral reads and reads with no hits) were then characterized through comparison to the Viral Refseq Protein NCBI database using Diamond with a maximum e-value of 0.001 and retaining only the top hit as the final viral classification.

In addition to analyses at the read level, the remaining reads (viral reads and reads with no hits) were then *de novo* assembled into contigs using the program SPAdes (Bankevich *et al.* 2012). Contigs were first compared to the non-redundant NCBI database using Diamond to remove other prokaryotic and eukaryotic matches, and then compared to the Viral Refseq Protein database, and contigs matching viral sequences were retained as a final set of viral contigs. An additional step in the pipeline predicts open reading frames (ORFs) in all contigs using the program getORF (part of EMBOSS; Rice *et al.* 2000), including those that have not been assigned to any known viral taxa, and which could represent new viral species or groups.
For reproducibility, the Diamond databases used for analyses (non-redundant, ViralRefSeq, and RefSeq protein) were standardized for samples that were compared to one another.

MOLECULAR ECOLOGY RESOURCES

Appendix S3: FBS sequencing

We analyzed two different batches of FBS (Gibco); sterile 100 µL aliquots of FBS were aliquoted to 96 well extraction plates along with 40 µL Proteinase K (Qiagen). Samples were inactivated at this stage by adding 600 µL of a mixture containing Buffer RLT, MagAttract Suspension G (both Qiagen) and isopropyl alcohol. All of these steps were performed according to CL2+ guidelines. Plates for wash steps were prepared; these included one plate containing 700 µL wash buffer AW1 and two plates containing 500 µL wash buffer RPE (both Qiagen). All plates were loaded onto a Kingfisher Flex 96 automated extraction machine (Thermo). The instrument settings, provided by Qiagen ('Protocol for purification of viral nucleic acid and bacterial DNA with Thermo Scientific KingFisher Flex'), consist of a lysis and binding step, followed by three wash steps, and a final elution in 80 µL Buffer AVE (RNase-free water with 0.04% NaN_3). Extracted nucleic acid was stored at -80°C.

For one aliquot, 10 µL nucleic acid was used directly as input into cDNA synthesis but for the other there was not sufficient nucleic acid in the 10 µL aliquot to prepare a library, so 40 µL was first concentrated to 10 µL using 1.8X Agencourt RNAclean XP beads (Beckman Coulter). cDNA synthesis was performed using the Maxima H Minus First Strand cDNA Synthesis Kit (Thermo) and converted to double strand cDNA using the NEBNext mRNA Second Strand Synthesis Module (New England Biolabs). Samples were library prepared using the KAPA DNA Library Preparation Kit for Illumina (KAPA Biosystems) modified for low input RNA samples.

Appendix S4: Mock viral swab extraction

Two extraction methods were initially tested: a magnetic bead-based extraction using the reagents of the Biosprint One-for-all-vet kit (Qiagen) and extraction using TRIzol reagent (Invitrogen). The TRIzol method, a guanidinium thiocyanate-phenol-chloroform extraction, was ineffective as no virus was detected by qPCR in extractions from any of the components of the sample (results not shown). This could be due to salt components of RNALater, an aqueous sulfate salt solution, which appeared to adversely affect the phase separation during the TRIzol extraction. Therefore, only the magnetic bead-based extraction method is further discussed. Reagents and volumes are based upon the manufacturer's protocol, and steps are a manual approximation of the automatic extraction method previously performed on the Kingfisher Flex 96 machine. Extractions were performed manually because the CVR, where the work was carried out, has a workflow which does not allow for lab propagated samples to be extracted on machines that are used for clinical or field collected samples, such as the Kingfisher.

Samples were thawed in a CL2 flow hood and the swab was removed into a tube containing 288 µL Buffer RLT and 40 µL Proteinase K. The swab/lysis buffer tubes were vortexed for 15 seconds, incubated for 5 minutes, vortexed again, and the swab removed. 100 µL DPBS was added to make up the volume required in the protocol.

The original tube without the swab was centrifuged at 13,000xg for 5 minutes. 100 µL supernatant was then removed to another tube containing 288 µL Buffer RLT and 40 µL Proteinase K. Finally, the remaining supernatant was removed from the original tube and the pellet resuspended in 100 µL DPBS. The resuspended pellet was transferred to a third tube containing 288 µL Buffer RLT and 40 µL Proteinase K.

To each of the three tubes now containing sample (swab, supernatant, resuspended pellet), lysis buffer and Proteinase K, 288 µL isopropanol and 24 µL MagAttract beads were added. Samples were mixed on a rotating tube mixer for 5 minutes, briefly spun down, beads pelleted using a magnetic bead separation rack and supernatant removed. All mixing and pelleting steps were performed in this way. Three wash steps were performed – 700 µL Buffer AW1 and 1 minute of mixing, followed by two steps of 500 µL Buffer RPE and 1 minute of mixing. After removing supernatant, beads were air dried for 15 minutes, 100 µL Buffer AVE was added and mixed for 5 minutes. Beads were pelleted and the supernatant was removed and kept. The extracted RNA was temporarily stored at -20°C before proceeding to cDNA synthesis.

cDNA synthesis was performed with 5 µL RNA using random primers and SuperScript III Reverse Transcriptase (Invitrogen) according to the manufacturer's instructions. The cDNA was then used as input into a qPCR assay to determine viral copy number in each extraction. The qPCR assay was performed using the Brilliant III Ultra Fast qPCR kit (Agilent) and previously designed

SBV primers and probe (Hoffmann *et al.* 2012) on an ABI7500 Fast Real Time PCR System (Applied Biosystems). qPCR reactions of samples and standards were run in triplicate, and quantity of viral copies was assessed against a standard curve of SBV concentrations ranging from 10^9-10^1 copies/µL. Controls containing no template were run in triplicate for each set of qPCR reactions. The approximate accuracy of the qPCR assay was confirmed by quantifying undiluted virus that had been extracted using the same method (concentration 3.9×10^5 PFU/mL or approximately 4.02×10^8 copies/mL) which was estimated as 2.2×10^8 copies/mL by qPCR.

MOLECULAR ECOLOGY RESOURCES

Appendix S5: Supplementary Tables

Table S1. Families and genera of vertebrate-infecting viruses. The list, which was compiled using the 2017 ICTV Taxonomy, was used to filter taxa lists for vertebrate-infecting viral taxa only for subsequent analyses.

**Family**	**Genus**
Hantaviridae	Orthohantavirus
Nairoviridae	Orthonairovirus
Peribunyaviridae	Herbevirus
	Orthobunyavirus
Phenuiviridae	Goukovirus
	Phlebovirus
Alloherpesviridae	Batrachovirus
	Cyprinivirus
	Ictalurivirus
	Salmonivirus
Herpesviridae	Iltovirus
	Mardivirus
	Scutavirus
	Simplexvirus
	Varicellovirus
	Cytomegalovirus
	Muromegalovirus
	Proboscivirus
	Roseolovirus
	Lymphocryptovirus
	Macavirus
	Percavirus
	Rhadinovirus
Bornaviridae	Bornavirus
Filoviridae	Cuevavirus
	Ebolavirus
	Marburgvirus
Nyamiviridae	Nyavirus
Paramyxoviridae	Aquaparamyxovirus
	Avulavirus
	Ferlavirus

	Henipavirus
	Morbillivirus
	Respirovirus
	Rubulavirus
Pneumoviridae	Metapneumovirus
	Orthopneumovirus
Rhabdoviridae	Curiovirus
	Ephemerovirus
	Hapavirus
	Ledantevirus
	Lyssavirus
	Novirhabdovirus
	Perhabdovirus
	Sprivivirus
	Sripuvirus
	Tibrovirus
	Tupavirus
	Vesiculovirus
Sunviridae	Sunshinevirus
Arteriviridae	Dipartevirus
	Equartevirus
	Nesartevirus
	Porartevirus
	Simartevirus
Coronaviridae	Alphacoronavirus
	Betacoronavirus
	Deltacoronavirus
	Gammacoronavirus
	Bafinivirus
	Torovirus
Picornaviridae	Ampivirus
	Aphthovirus
	Aquamavirus
	Avihepatovirus
	Avisivirus
	Cardiovirus
	Cosavirus
	Dicipivirus

	Enterovirus
	Erbovirus
	Gallivirus
	Harkavirus
	Hepatovirus
	Hunnivirus
	Kobuvirus
	Kunsagivirus
	Limnipivirus
	Megrivirus
	Mischivirus
	Mosavirus
	Oscivirus
	Parechovirus
	Pasivirus
	Passerivirus
	Potamipivirus
	Rabovirus
	Rosavirus
	Sakobuvirus
	Salivirus
	Sapelovirus
	Senecavirus
	Sicinivirus
	Teschovirus
	Torchivirus
	Tremovirus
Adenoviridae	Atadenovirus
	Aviadenovirus
	Ichtadenovirus
	Mastadenovirus
	Siadenovirus
Anelloviridae	Alphatorquevirus
	Betatorquevirus
	Deltatorquevirus
	Epsilontorquevirus
	Etatorquevirus
	Gammatorquevirus

	Gyrovirus
	Iotatorquevirus
	Kappatorquevirus
	Lambdatorquevirus
	Thetatorquevirus
	Zetatorquevirus
Arenaviridae	Mammarenavirus
	Reptarenavirus
Asfarviridae	Asfivirus
Astroviridae	Avastrovirus
	Mamastrovirus
Birnaviridae	Aquabirnavirus
	Avibirnavirus
	Blosnavirus
Caliciviridae	Lagovirus
	Nebovirus
	Norovirus
	Sapovirus
	Vesivirus
Circoviridae	Circovirus
	Cyclovirus
Flaviviridae	Flavivirus
	Hepacivirus
	Pegivirus
	Pestivirus
Genomoviridae	Gemycircularvirus
	Gemygorvirus
	Gemykibivirus
	Gemykolovirus
	Gemykrogvirus
	Gemykroznavirus
	Gemytondvirus
	Gemyvongvirus
Hepadnaviridae	Avihepadnavirus
	Orthohepadnavirus
Hepeviridae	Orthohepevirus
	Piscihepevirus
Iridoviridae	Lymphocystivirus

	Megalocytivirus
	Ranavirus
Nodaviridae	Alphanodavirus
	Betanodavirus
Orthomyxoviridae	Influenzavirus A
	Influenzavirus B
	Influenzavirus C
	Influenzavirus D
	Isavirus
	Quaranjavirus
	Thogotovirus
Papillomaviridae	Alphapapillomavirus
	Betapapillomavirus
	Chipapillomavirus
	Deltapapillomavirus
	Dyochipapillomavirus
	Dyodeltapapillomavirus
	Dyoepsilonpapillomavirus
	Dyoetapapillomavirus
	Dyoiotapapillomavirus
	Dyokappapapillomavirus
	Dyolambdapapillomavirus
	Dyomupapillomavirus
	Dyonupapillomavirus
	Dyoomegapapillomavirus
	Dyoomikronpapillomavirus
	Dyophipapillomavirus
	Dyopipapillomavirus
	Dyopsipapillomavirus
	Dyorhopapillomavirus
	Dyosigmapapillomavirus
	Dyotaupapillomavirus
	Dyothetapapillomavirus
	Dyoupsilonpapillomavirus
	Dyoxipapillomavirus
	Dyozetapapillomavirus
	Epsilonpapillomavirus
	Etapapillomavirus

	Gammapapillomavirus
	Iotapapillomavirus
	Kappapapillomavirus
	Lambdapapillomavirus
	Mupapillomavirus
	Nupapillomavirus
	Omegapapillomavirus
	Omikronpapillomavirus
	Phipapillomavirus
	Pipapillomavirus
	Psipapillomavirus
	Rhopapillomavirus
	Sigmapapillomavirus
	Taupapillomavirus
	Thetapapillomavirus
	Treisdeltapapillomavirus
	Treisepsilonpapillomavirus
	Treisetapapillomavirus
	Treiszetapapillomavirus
	Upsilonpapillomavirus
	Xipapillomavirus
	Zetapapillomavirus
Parvoviridae	Amdoparvovirus
	Aveparvovirus
	Bocaparvovirus
	Copiparvovirus
	Dependoparvovirus
	Erythroparvovirus
	Protoparvovirus
	Tetraparvovirus
Picobirnaviridae	Picobirnavirus
Polyomaviridae	Alphapolyomavirus
	Betapolyomavirus
	Deltapolyomavirus
	Gammapolyomavirus
Poxviridae	Avipoxvirus
	Capripoxvirus
	Centapoxvirus

	Cervidpoxvirus
	Crocodylidpoxvirus
	Leporipoxvirus
	Molluscipoxvirus
	Orthopoxvirus
	Parapoxvirus
	Suipoxvirus
	Yatapoxvirus
Reoviridae	Orbivirus
	Rotavirus
	Seadornavirus
	Aquareovirus
	Coltivirus
	Orthoreovirus
Retroviridae	Alpharetrovirus
	Betaretrovirus
	Deltaretrovirus
	Epsilonretrovirus
	Gammaretrovirus
	Lentivirus
	Spumavirus
Togaviridae	Alphavirus
	Rubivirus
Deltavirus	Deltavirus
Tilapinevirus	Tilapinevirus

Table S2. Bovine viral diarrhea virus nucleotide blast hits. Blast results from large contigs assembled from FBS samples. Contig length and k-mer coverage are reported based on the SPAdes assembly. Query coverage and percent identity are from the nucleotide blast, in addition to accession number and description of the top blast hit from the Genbank nt database.

**Contig ID†**	**Contig Length (bp)**	**K-mer coverage**	**Blast Genbank ID**	**Blast Query cover**	**Blast Identity (%)**	**Description of blast hit**
FBS1 NODE_57	1396	3.33	KY683847.1	100%	96%	Bovine viral diarrhea virus 3 strain SV757/15
FBS1 NODE_74	1280	2.49	KY683847.1	100%	97%	Bovine viral diarrhea virus 3 strain SV757/15

FBS1 NODE_99	1165	4.07	KY683847.1	100%	97%	Bovine viral diarrhea virus 3 strain SV757/15
FBS2 NODE_244	775	1.37	KY683847.1	100%	98%	Bovine viral diarrhea virus 3 strain SV757/15

†Contig sequences are provided in Table S3

Table S3. Contig sequences matching bovine viral diarrhea virus detected in FBS. Contig names correspond to those in Table S2.

**Contig ID**	**Sequence**
FBS1 NODE_57	CCTGATGTTCTCCAGCTTGGTCAGAGGTGATGATGGGAGGGTGTCCCAAGGAGATGAACT<br>TGACGTATGGTTTTATGTGGTATTGGCTATGGTATGCATACTCCTGATGGTAAAAAGAGA<br>CCCCACAACAATCCCTGCAGTAGTGATAATAACAGGTGTTAAAACAAGACAATATGCTGC<br>ATGGCTAGAACTAGATGTAGCTTTGTCAATAGTTGCGGCCATTGTACTACTCCATTCCTA<br>TATTAGTAGTTACTATAGATACAAACAATGGTTACAGTGTGTGATAAGTCTACTAGCTGG<br>TTTTTTTATAATAAGGACGTTGAAAGCTGTAGGGGAGTGCCAACTGCCTGTAATAACAGT<br>GCCTAATGTGAAGCCCTTGCCCATAGTGATTATATACCTAATTACAACCACTTTGGTGAC<br>CCACCAGAACCTGGATTTGGCTGGAATATTCCTCAGTAACGCACCAATTATGTTAATGGT<br>ACTAACCCTGTGGGCAGATCTACTTACCCTCATTCTGGTCCTGCCAACTTATGAATTAAC<br>AAAATTGTACTACATCAGAAGGGTGAAGAAGGACGTAGAGAGGAGTTGGCTAGGGGCTAC<br>AAACTTCACACGTGTGGACTCTGTGTATGAATTAGATGGATCTGAAGAAGGGGTGTATTT<br>ATTCCCCTCCAGGCAAGGCCCCGGAGCAAAAACAGGTGAAATACTACCAGTATTGAGGTG<br>TGTGTTAATAAGCTGCGTCAGCAGTTACTGGCAGTGGACATATCTAATCTACCTGGTGAT<br>TGAATTGGTATATTTCATGCATAGAAGAGTGATAGAAGAGGTAGCTGGTGGAACCAACGC<br>GCTCTCTAGAATGATTGCTGGGCTGATAGAAATGAGTTGGGCTCTGGACGAAGAAGAGGG<br>CAGGGGGCTTAAAAAGTTCTACATTTTGTCTGCTAGGCTCAAGAATTTGGTAATGAAGCA<br>CAAGGTCAGAAATGAAACTATAAGGGCGTGGTATGAGGAAGAAGAAATTTATGGAATGCC<br>AAAAGTTGTCACATTGATCAAAGCTGCCTCATTAAGCCAGAGCAAACACTGTATTCTATG<br>CACTGTATGTGAAAGACGTGATTGGAAGGGTGGTAGCTGCCCTAAATGCGGTCGTTCCGG<br>CAGGCCCATATCATGCGGAATGACTCTAGCTGACTTTGAGGAAAAACATTATAAAAGGAT<br>CTTCATAAGAGAGGGAGAATTGGACGGGCCTTTCAGGCAGGAGGCCAGAGGGTATTTACA<br>ATACATAGCAAGAGGACAGCTTTTCCTAAGGAATTTACCAATACTGGCAACCAAAGTAAA<br>ACTATTAATGGTAGGGAACCTGGGATCCGAAGTAGGCGATCTCGAACACCTAGGTTGGGT<br>TCTGAGGGGACCTGCG
FBS1 NODE_74	CTGACTCTTTTCTCACACTTCAGTCCTGTGTCTACCACTACATCTAGATCAGGTAAGGTC<br>ACTCCTGATTCTATGGCGTTTGTGGCAACTACTACATATGGTGATTGTGATGTAACAACC<br>CTCAGGTTTGCTGGGTCCTCTCCACTATAATAATAGCCTGAGTTGTAACCTTTAGCCTTC<br>AATTTCTTTGCCACCTCCACAGCCATATTCCTAGTGGGTACAAACACTAACATATTACCC<br>TTCATTTCTTCTGTTGGTATTTTAAGTCCAGCTATATCCAGGAAATTTGATCCCAGATCC<br>TCCCCCTTCATAACTTCTGGAGCTATAAACTCCTCGATAGGGTGTTTCTGCCCAGTGGTT<br>GTGACTGACCCAGCTGGAGTAGCGGTCATAGCCACAACTCTTAGGTTTTCCGAGAACCTG<br>TGGATCTTCCCTATTATGGCCAACTGCTCCGGAGTAGCACAGTGGTATTCATCCAGGAAG<br>ATATAAGAGTATTCCACCATTGCTGCTCTCAATTTTGGCTGGGGCATTTGGCAAAAGTAT<br>CCGTATGAGGCATATGTTATCCCTGTAGCCATGTCACCTTCTTTCATTTCCCCTATCCTG<br>AGGTTGAAGGCAATACTGGGGTGCTTTTGTTTCATATACTGGTATACTGATTCCGCCGCT

	GCTCGCAGCGGGATTAGAACCAATACCCTCTTGTGTCTTCCAATTTCTTCTATCACTGAT<br>CTTGGAAGCTCTGTGGTTTTTCCTGCCCCCGTGGCCAATGTTATTTGCTTGAATTCCCCC<br>CTGTTCATTGCTACTATTTTCTTTACCATCTCTGTCAAGTCAGTTGTGCTTTTTGACACT<br>GTTTGTATCCCACTCATAAGCTTGGTGGGTTTGGACTCTTCATTCTTCCCAACTTTGACT<br>CTCCCAACAACCCTGCCGCTGGATGCTTCGAATATGGGCAATCCGGACCAGCCTTTTAAG<br>TTCTTCAGGTCGAAGAAAGCTGGAGTGCCTGAGGCAGTCACACAGGTGAACTCCCCTCCT<br>GTTTTCTGCAGATGTACCATTGCTCCTCTGGTCCCAGCTATGTTCTGTGCCTCTGGATTC<br>AGCACGTAACACCTAGCGCCTTCTGGACAACCTGAATCAGTCTTTATGCCATATTCGGTC<br>TCATCTGTCATTTTGTTGTTACTTTGGCACACCACCCTGGTTCGGCCTAATGAGTCACAG<br>ACCAGCAGGTCTTTCCCTGCCGTTACATGGTCCACTGAACTTATCCCCCCTTGGTGTGTA<br>TAAGCCCATCCTGTTTCCAG
FBS1<br>NODE_99	TGCTGTTAATTAATGAGGTAGTGTTGTTGAATGAGGTAGTTCTCATTAGCCTAAAGTAAT<br>AAATTAGCTCTGACAATACCTACACTATATATTACATGTTATTGTTATTTACATTTATAT<br>ACACACATGTTCCTGCCTAGATGACAGCCTTCTCTCCTATGGCCGCCATCATCAAGACCC<br>TCAGCCTTCTCAGGATTACATTGACTATTGGACCAAGACGGTATCTGTCTTTCCCCACCC<br>TTAAGATGGGTCCTCCTGGCCCTATTAGCCTCAGTTCTTCATAATACTTGCCTTGCAAGG<br>TGTGTCCTTCATCGGGGATGTACATTTTGTTGGTCTTACTAGAAACCAGCCGGTCAGCAT<br>TGACAAGCCAGTTACCTTCTTCTTTCCCCACCTTCACACAGTCTTCGAGTAGTCTTTTAC<br>TAGTGTGCCTTGTCCATATCCCCAATGTGGACATACTCAGGTTTAAGCTAGCCAGCTTCT<br>CAAAACCAACTCTCTTCAGATCATGCAGGTTGTGCCCTACAACCTCCTTGTAGGCCCCTA<br>TAGGGTCTCCCTTATAGTAGTAAGTGGCTTGTTTAGAAGGGTCTACCTCTGGTTTTGATG<br>ATAAGGTCAACAGACAAATCCTCCTGATTAAAGGGTTCCAGGAGTACATCAGGAGGAAGC<br>TGAATGCCACTGCTTTCTCATATGCGGCAGTGCCCCTCTCCCCACTTGAGTCAAGCCTAG<br>TGGCCATTTTTGCAAGTACTGTGGCGGTGTTTCTTCCGGCCATGTAGCCGCTGCTGTTGT<br>CTGACCATCTAACGGGGATTGGTGTGTGGGAACAAAACTCTATGTCTTCAAATCTGTAAG<br>CCACCTTCATTTTGTCTCCTTCTAGTATCTTTTGTGGTTTGCCAGATTCATACAATATCT<br>GGGCTCCTTTACTGGCAAACTTCAACCCCAGTCCTCTCTCTGTTATCAGAAAGCCGTCGT<br>CTCCACAGACATGTATTCTTGCTACTCTGTTGAAACTCCTGTACGGGACCCCAGTGCTCT<br>CACAAAAAGCATACACCATGGTTAGCACATTTAGCATGCTGTTCCCTGCGCTTGTGTCTG<br>GTTGGCCACTGCCCCTCTGCCCCTTTCTTATGTAGACCTCACCATCAGCTGTGATCACCG<br>GGACCTCAGTCATGTGTTCTGTTAA
FBS2<br>NODE_244	CACCATGGTTAATACATTCAGCATGCTGTTCCCTGCACTTGTATCTGGTTGGCCACTGCC<br>CCTCTGCCCCTTTCTTATGTAGACCTCACCATCAGCTGTGATTACCGGGACCTCAGTCAT<br>GTGTTCTGTTAAGGTGTCAATGAACTTATGCCATCTGCTCTTGAAATAGTATTTTTGTAT<br>CCTCCCTATGAGGCGGAGATCGCGGCTCGTCACCTGAGTGTCCCAAGCCTTTGTGTCAAA<br>ACTCACTGCCACCGGGTCGTTGAAGCCATCCCACTCCTTCTTTACCTTGTCAAAGATTTG<br>GAAAAGTGGGGTTTTTCCTTCGTACCCCGGTATCACCACAGGTTTTTGCTTGACCCACTT<br>GTACATCACTTTGGTGATTGCCAATCTGACTTTGGCATCTGGGTACTGTATAACTCTTGG<br>TTTCTTTTCATCAACCAAGTCCCCTGCTTCCCAGTCATCGTTGACATCTCTCTTCTCATT<br>CTTAGGTATGGCTGTCTCATAGTAATTGATCTTCTTCCCTCTTTTCAGGTCCTTGACTAT<br>TTCTTCAACTAAGTTCTTCTCTGAAGCGAGCACCTCCCCTATATTTTTCTTTTCCAGGAA<br>CCCTGCTGCTCCTTTCCTGTTTATGCCAGCCTCTAGTTCCTCCCATGACACCTCGTCATA<br>AACACCTTTTAGTTCTGGGACCTTAAGCGTATCAAACACTTCCCATAGCTTCTCATGCAG<br>ATCTGGGCTTTGGGCGTTCTCTTCCTTATCTATTTTGTCTCTGATTGCTTGATGG

MOLECULAR ECOLOGY RESOURCES

Table S4. Model comparison between linear and polynomial models for each sample type (feces and saliva) and filtering (all viral genera and vertebrate-infecting only) combination at the family level. For each combination, two models were run and compared through both likelihood ratio test (*L*, X^2, d.f., and *P*-value) and AIC (AIC and ΔAIC).

Sample	Model	*L*	X^2	d.f.	*P*-value	AIC	ΔAIC
All Genera Fecal	Linear	-590.54	31.59	1	1.90E-08	1187.1	29.591
	Polynomial	-574.75				1157.5	
All Genera Saliva	Linear	-847.37	42.641	1	6.58E-11	1700.7	40.641
	Polynomial	-826.04				1660.1	
Vert Genera Fecal	Linear	-400.72	8.8299	1	0.002963	807.45	6.83
	Polynomial	-396.31				800.62	
Vert Genera Saliva	Linear	-715.48	15.786	1	7.09E-05	1437	13.786
	Polynomial	-707.59				1423.2	

MOLECULAR ECOLOGY RESOURCES

Appendix S6: Supplementary Figures

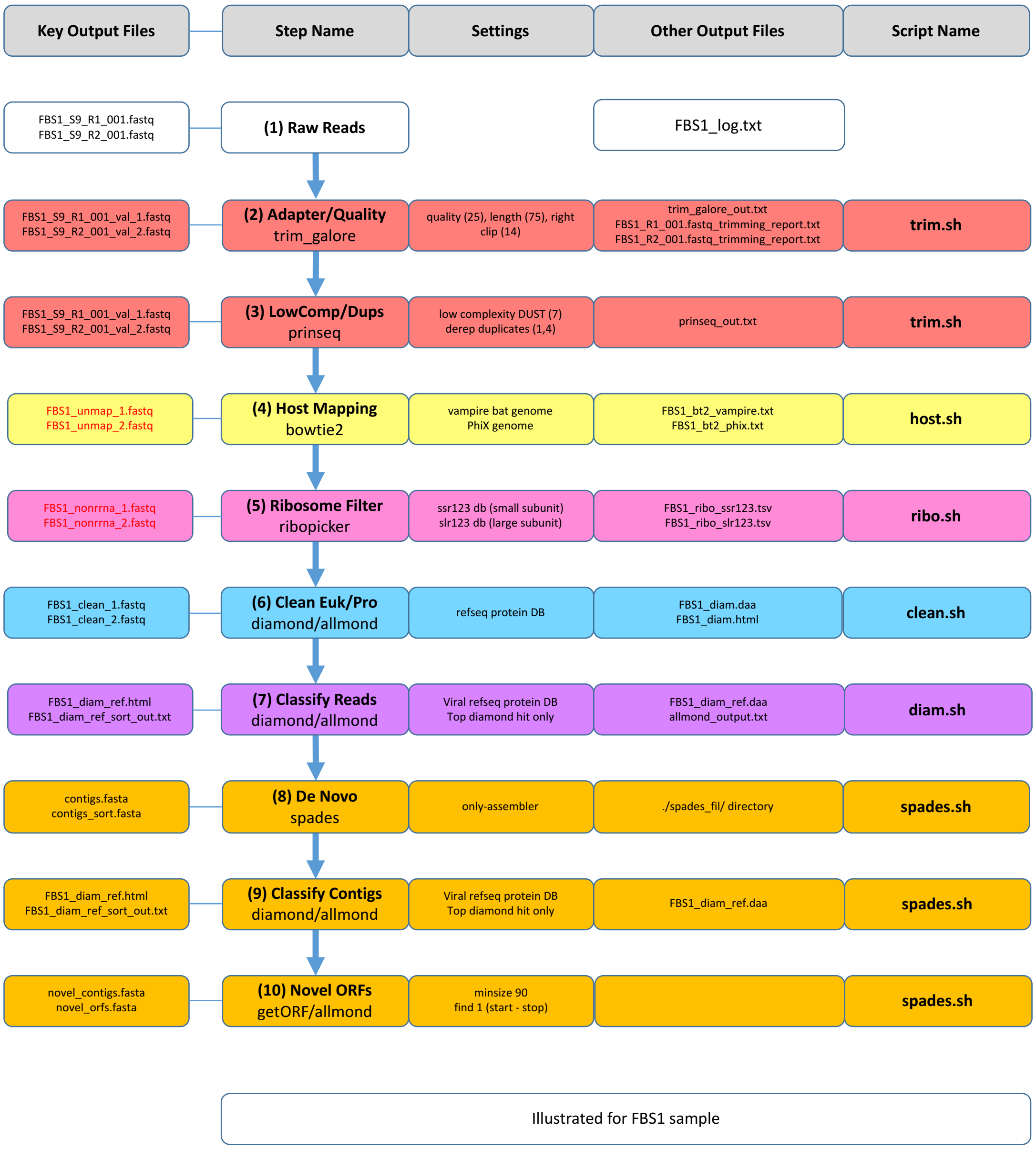

Figure S1. Schematic diagram of the bioinformatic pipeline developed to analyze vampire bat viral communities. The diagram depicts each step, the script used to perform the analysis, settings used, and output files (using sample FBS1 as an example).

MOLECULAR ECOLOGY
RESOURCES

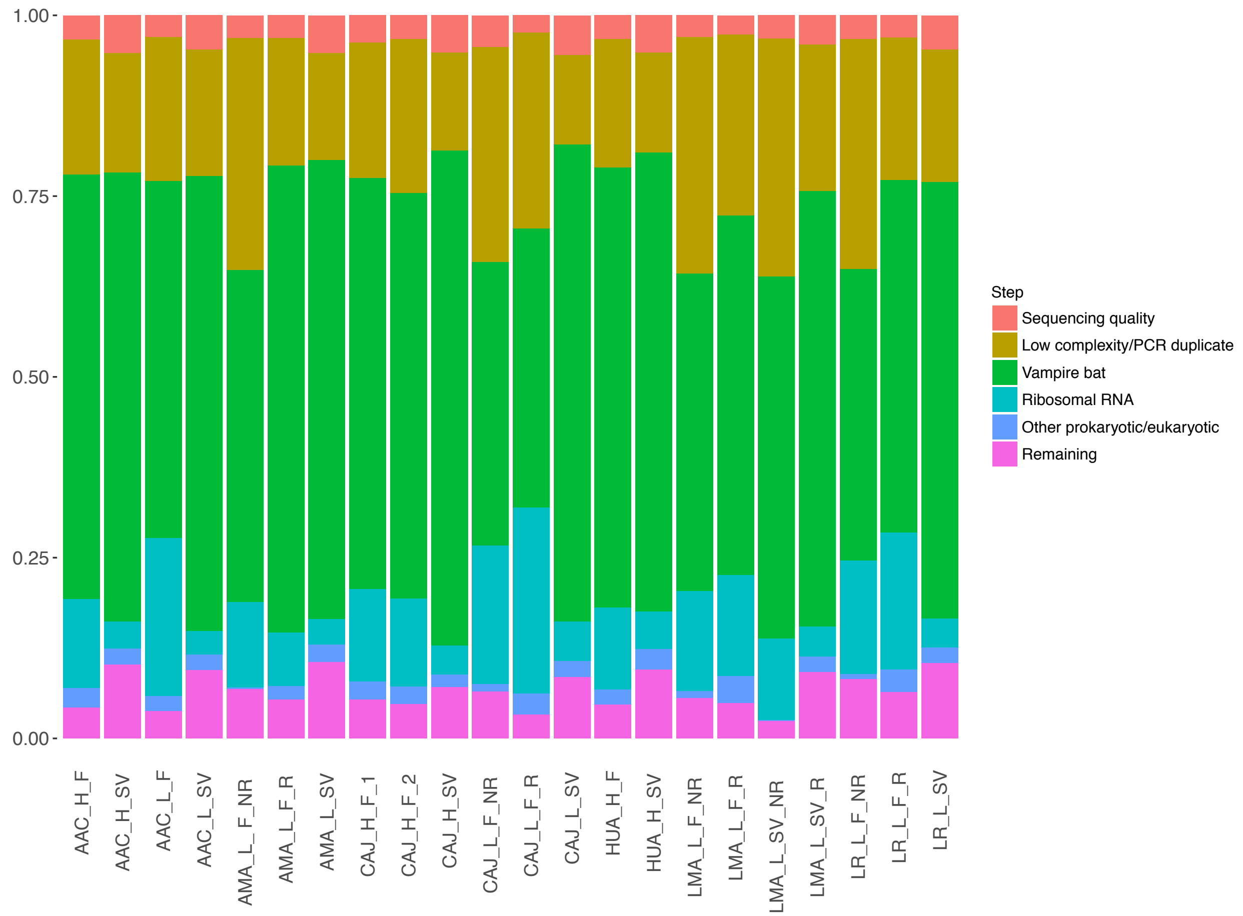

Figure S2. Proportion of reads filtered out during bioinformatic processing steps from multi-colony pools (names correspond to Table 1). Pools were processed using the viral community bioinformatic pipeline. Sequencing quality reads are those removed by Trim Galore, low complexity/duplicate reads are removed by prinseq-lite, vampire bat are reads mapping the vampire bat genome, PhiX are reads mapping to the PhiX genome, Ribosomal RNA are those removed by RiboPicker, and other prokaryote/eukaryote are reads assigned to those taxa when using Diamond to compare reads to the Genbank nr database. Remaining reads are those assigned to viruses or unassigned.

MOLECULAR ECOLOGY RESOURCES

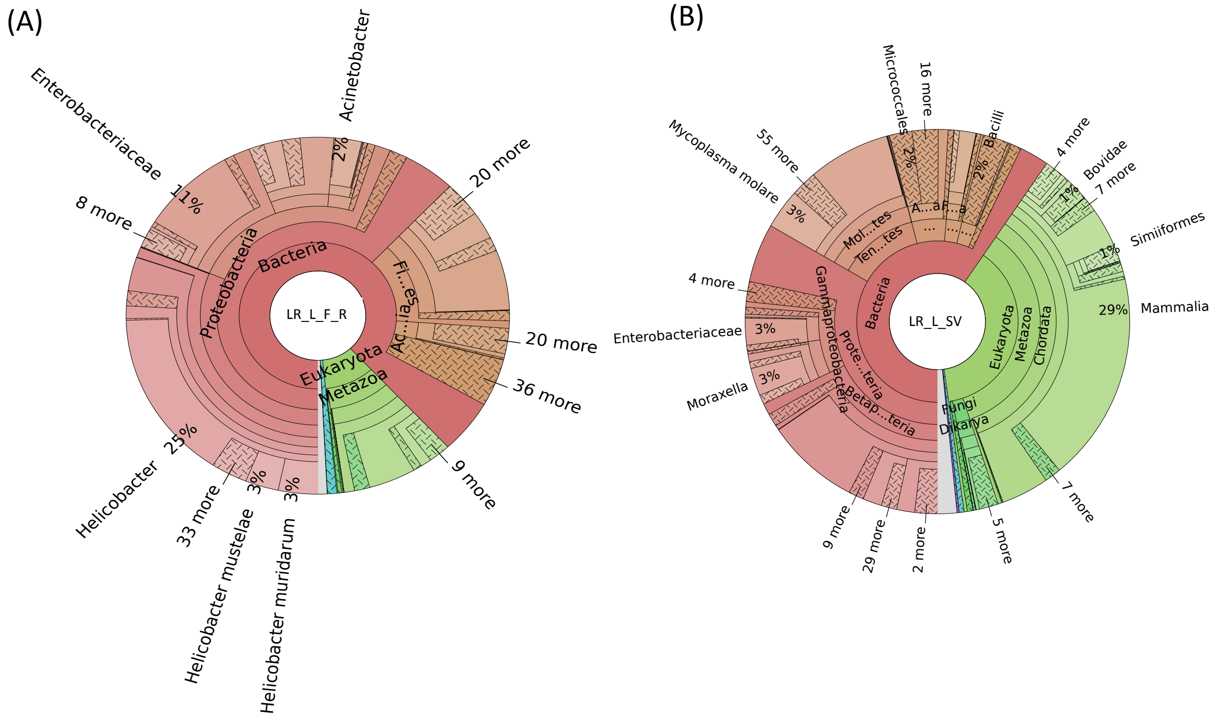

Figure S3. Krona plots depicting the distribution of reads from locality LR in (A) fecal and (B) saliva pools which were processed according to the optimized protocol. Reads are shown following quality filtering, rRNA depletion, and host subtraction but prior to subtraction of reads closely matching other prokaryotic/eukaryotic taxa based on Diamond blast comparison to the Genbank nr database. Red segments are bacterial taxa and green are eukaryotic taxa, while archaea and viruses are represented by smaller blue and purple segments respectively, and unassigned taxa are shown in gray. Taxa with a high percentage of reads out of the total have names shown, while names of taxa with lower percentages are not depicted to facilitate visualization. Sample names are shown inside plots and correspond to descriptions in Table 1.

MOLECULAR ECOLOGY RESOURCES

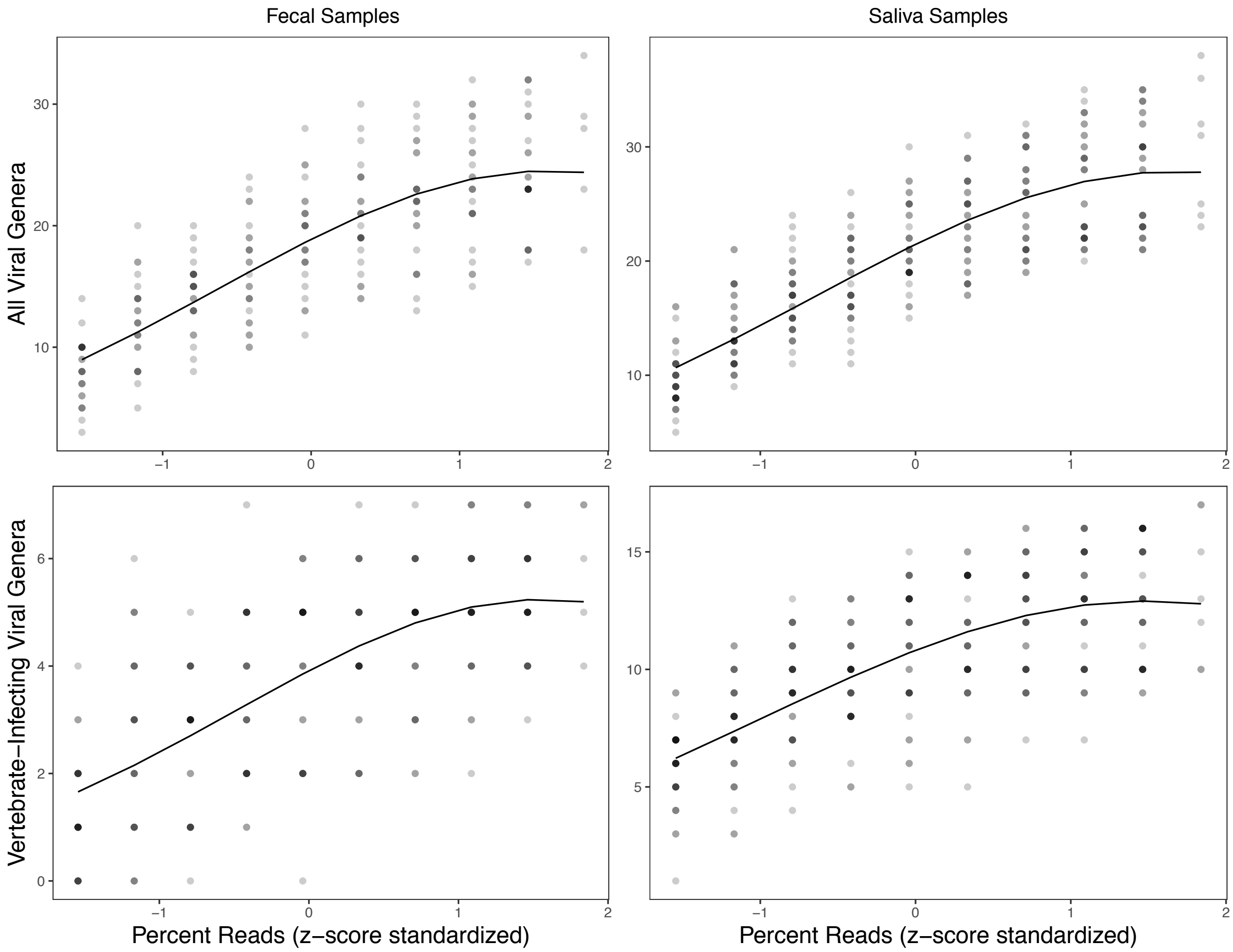

Figure S4. Number of viral genera and vertebrate viral genera detected in fecal (N=5) and saliva (N=7) samples at increasing percentage of the original raw reads. Percent reads are z-score standardized by subtracting the mean and dividing by the standard deviation. Points, which are semi-transparent to indicate density, show the rescaled original data, and lines show the model prediction.

Appendix S7: Supplementary References

Andrews S (2010) FastQC: a quality control tool for high throughput sequence data.

Bankevich A, Nurk S, Antipov D *et al.* (2012) SPAdes: A New Genome Assembly Algorithm and Its Applications to Single-Cell Sequencing. *Journal of Computational Biology*, **19**, 455–477.

Buchfink B, Xie C, Huson DH (2014) Fast and sensitive protein alignment using DIAMOND. *Nature Methods*, **12**, 59–60.

Hoffmann B, Scheuch M, Hoeper D *et al.* (2012) Novel Orthobunyavirus in Cattle, Europe, 2011. *Emerging infectious diseases*, **18**, 469–472.

Langmead B, Trapnell C, Pop M, Salzberg SL (2009) Ultrafast and memory-efficient alignment of short DNA sequences to the human genome. *Genome Biology*, **10**, R25.

Martin M (2011). Cutadapt removes adapter sequences from high-throughput sequencing reads. *EMBnet.Journal,* **17**, 10-12.

Mukherjee S, Huntemann M, Ivanova N, Kyrpides NC, Pati A (2015) Large-scale contamination of microbial isolate genomes by Illumina PhiX control. *Standards in Genomic Sciences*, **10**, 18.

Rice P, Longden I, Bleasby A (2000) EMBOSS: The European molecular biology open software suite. *Trends in Genetics*, **16**, 276–277.

Schmieder R, Edwards R (2011) Quality control and preprocessing of metagenomic datasets. *Bioinformatics*, **27**, 863–864.

Schmieder R, Lim YW, Edwards R (2012) Identification and removal of ribosomal RNA sequences from metatranscriptomes. *Bioinformatics*, **28**, 433–435.

Zepeda-Mendoza, M.L. et al. (2018) Hologenomic adaptations underlying the evolution of sanguivory in the common vampire bat. *Nature Ecology & Evolution*, **2,** 659-668.
